# Current Strategies for the Regeneration of Skeletal Muscle Tissue

**DOI:** 10.3390/ijms22115929

**Published:** 2021-05-31

**Authors:** Emine Alarcin, Ayca Bal-Öztürk, Hüseyin Avci, Hamed Ghorbanpoor, Fatma Dogan Guzel, Ali Akpek, Gözde Yesiltas, Tuba Canak-Ipek, Meltem Avci-Adali

**Affiliations:** 1 Department of Pharmaceutical Technology, Faculty of Pharmacy, Marmara University, 34854 Istanbul, Turkey; emine.alarcin@marmara.edu.tr; 2 Department of Analytical Chemistry, Faculty of Pharmacy, Istinye University, 34010 Istanbul, Turkey; aozturk@istinye.edu.tr; 3 Department of Stem Cell and Tissue Engineering, Institute of Health Sciences, Istinye University, 34010 Istanbul, Turkey; 4 Department of Metallurgical and Materials Engineering, Eskisehir Osmangazi University, 26040 Eskisehir, Turkey; havci@ogu.edu.tr; 5 Cellular Therapy and Stem Cell Research Center, Eskisehir Osmangazi University, 26040 Eskisehir, Turkey; 6 AvciBio Research Group, Eskisehir Osmangazi University, 26040 Eskisehir, Turkey; hamed.ghorbanpoor@ogu.edu.tr; 7 Translational Medicine Research and Clinical Center, Eskisehir Osmangazi University, 26040 Eskisehir, Turkey; 8 Department of Biomedical Engineering, Ankara Yildirim Beyazit University, 06010 Ankara, Turkey; fdogan@ybu.edu.tr; 9 Department of Biomedical Engineering, Eskisehir Osmangazi University, 26040 Eskisehir, Turkey; 10 Department of Bioengineering, Gebze Technical University, 41400 Gebze, Turkey; aliakpek@gtu.edu.tr (A.A.); gyesiltas@gtu.edu.tr (G.Y.); 11 Department of Thoracic and Cardiovascular Surgery, University Hospital Tuebingen, Calwerstraße 7/1, 72076 Tuebingen, Germany; tuba.canak-ipek@uni-tuebingen.de

**Keywords:** skeletal muscle cells, tissue engineering, hydrogels, scaffold topographies

## Abstract

Traumatic injuries, tumor resections, and degenerative diseases can damage skeletal muscle and lead to functional impairment and severe disability. Skeletal muscle regeneration is a complex process that depends on various cell types, signaling molecules, architectural cues, and physicochemical properties to be successful. To promote muscle repair and regeneration, various strategies for skeletal muscle tissue engineering have been developed in the last decades. However, there is still a high demand for the development of new methods and materials that promote skeletal muscle repair and functional regeneration to bring approaches closer to therapies in the clinic that structurally and functionally repair muscle. The combination of stem cells, biomaterials, and biomolecules is used to induce skeletal muscle regeneration. In this review, we provide an overview of different cell types used to treat skeletal muscle injury, highlight current strategies in biomaterial-based approaches, the importance of topography for the successful creation of functional striated muscle fibers, and discuss novel methods for muscle regeneration and challenges for their future clinical implementation.

## 1. Introduction

Skeletal muscle is the most abundant tissue in the human body, accounting for approximately 40–45% of total body mass [1]. It is a form of striated muscle tissue that is under the voluntary control of the somatic nervous system and is primarily responsible for generating a series of discrete uniaxial forces that enable locomotion. Fortunately, skeletal muscle tissue has a high potential for self-repair after injury. However, severe injuries involving volumetric muscle loss (VML) result in permanent functional limitations [2]. Since VML cannot be restored naturally, it requires interventional treatment. It can significantly affect patients’ quality of life by severely limiting musculoskeletal functionality. Skeletal muscle damage is mainly caused by traumatic injuries, tumor resections, and degenerative genetic diseases, such as Duchenne muscular dystrophy (DMD), amyotrophic lateral sclerosis (ALS), and pediatric Charcot–Marie–Tooth disease, leading to muscle fiber atrophy.

Skeletal muscle consists of hierarchically organized muscle fibers, also known as myofibres, blood vessels, nerves, and connective tissue. Myofibers are formed by the fusion of myoblasts into elongated, cylindrical, multi-nucleated fibers named myotubes, which are 10–100 µm in diameter, depending on the location and function of the muscle [3]. They represent the functional unit of skeletal muscle. Each muscle fiber is a skeletal muscle cell and consists of multiple myofibrils with repeating sarcomeres, the contractile unit of skeletal muscle. The sarcomeres contain the contractile proteins thin filament actin and thick filament myosin. The muscle fiber is surrounded by the plasma membrane, the sarcolemma, and a cytoplasm named the sarcoplasm. As myofibers mature, their nuclei orient along the periphery below the sarcolemma. In skeletal muscle tissue, parallel myofibers are bundled into fascicles enclosed by the perimysium. For the contraction, each muscle fiber is supplied by a somatic motor neuron. The presence of many nuclei enables the production of large amounts of proteins and enzymes necessary for the proper function of these large, protein-rich cells.

Muscle regeneration relies on a heterogeneous population of resident muscle stem cells, also named satellite cells (SCs), interstitial cells, and blood vessels, and is mainly controlled by extracellular matrix (ECM) proteins, and secreted factors [4]. The field of tissue engineering aims to repair or regenerate lost or damaged skeletal muscle tissue through a combination of cells, scaffolds, and growth factors. Three-dimensional (3D) biomaterial scaffolds can be used to locally control and provide guidance for tissue regeneration. These scaffolds offer an artificial ECM for cells and mimic the properties of tissue. Furthermore, bioactive agents, such as proteins and growth factors, can be incorporated into scaffolds to support cell adhesion and proliferation and promote the synthesis of the cell’s own ECM. Scaffolds have been designed from a variety of established and novel biomaterials to provide them with desired mechanical, structural, physicochemical, and biological properties. This has led to a range of multifunctional biomaterials that can fulfill several important functions, such as cell guidance [5], drug delivery [6], and stem cell fate regulation [7].

## 2. Strategies for Skeletal Muscle Regeneration

**Figure 1:**
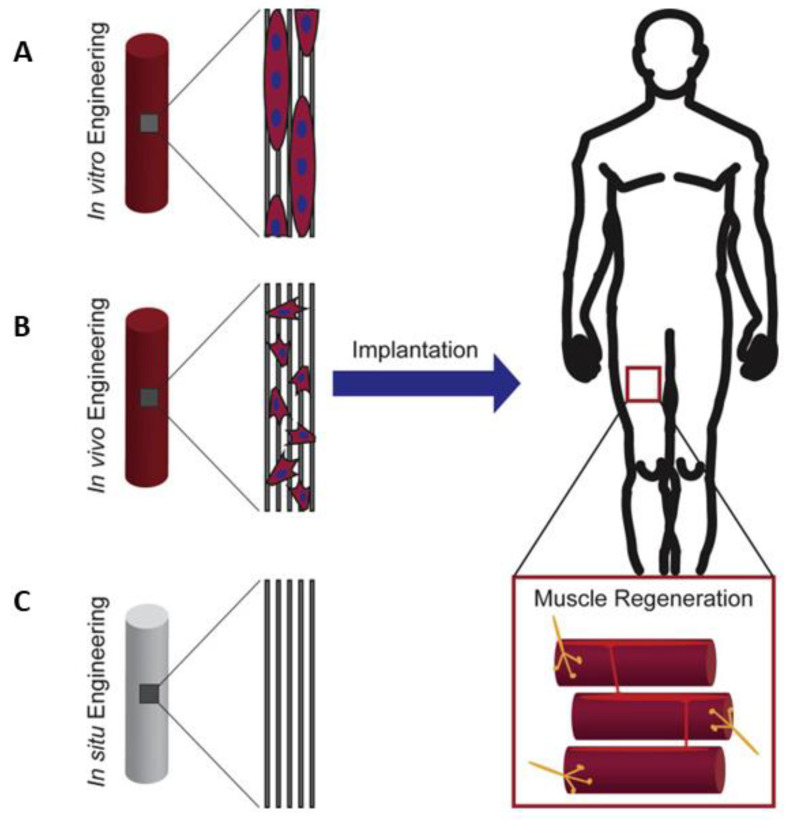
Demonstration of scaffold-based tissue engineering strategies for regeneration of skeletal muscle. (**A**) In vitro engineering uses cell-loaded preconditioned constructs to improve cell viability/survival, integration of the graft, and reinnervation. (**B**) In vivo engineering employs cell-laden biomaterials without extensive preconditioning. The differentiation should occur at the injury site after the transplantation. (**C**) In situ engineering uses acellular structures as its operating principle. The structure and biochemical properties of biomaterials enhance the infiltration of cells and regeneration of the tissue. Reprinted from [8] with permission from Elsevier.

The ability of functional striated muscle structures to integrate into the defective region of the host is one of the advantages of the skeletal muscle engineered constructs, which can enable the regeneration of the muscle [8]. In recent years, in vitro, in vivo, and in situ muscle engineering approaches have been applied to produce and regenerate skeletal muscle (Figure 1). Each approach has its own advantages and disadvantages [9]. The in vitro skeletal muscle engineering approach generally includes the in vitro differentiation of cells into myotubes [10]. The tissue structure is optimized by preconditioning in a bioreactor before implantation. This strategy aims to generate mature contractile functional constructs for transplantation. In addition, often a coculture of cells or multipotent cells is used to facilitate and support the formation of blood vessels and neuromuscular junctions (NMJs) [11]. However, the main challenges of in vitro skeletal muscle tissue engineering are the required dimension, complexity of the structure, insufficient alignment of myotubes, insufficient contractile force generation [1], and the high cell density required to induce differentiation into aligned myotubes. Thus, it is still a challenge to obtain large constructs for VML repair [12]. In addition, successful vascularization is an important parameter to ensure nutrient and oxygen supply to highly metabolically active cells in large constructs. Moreover, the biomaterials selected for in vitro skeletal muscle engineering have to be biocompatible and the applied cells should not cause rejection reactions.

The in vivo strategy relies on the transplantation of cells with or without scaffold to generate a local niche at the transplantation site [13]. This strategy aims to stimulate the regeneration of muscle, vasculature, and myoneural junctions in vivo. In vivo engineering bypasses the excessive treatment of cell-loaded constructs to ensure the functional properties of the cells to promote the new tissue formation in vivo. The main advantages of this system are reduced complexity and costs [1].

Acellular biomaterials are used as guides for in situ engineering strategies to stimulate endogenous regeneration of skeletal muscle. These biomaterials can be designed to provide specific topographies, release bioactive and chemotactic molecules to attract, activate, and stimulate the infiltration of cells from the injury site and induce muscle, vasculature, and NMJ regeneration [8]. The development of the in situ engineering strategy depends on advances in biomaterial science and tissue engineering, which also include scaffold fabrication techniques [8,14]. This strategy has the main advantage that it can enable the production of an off-the-shelf product for clinicians. However, this approach also requires complex biomaterials and extensive studies to understand the interactions between the biomaterial and the healing responses.

## 3. Cell Types for the Regeneration of Skeletal Muscle

**Figure 2:**
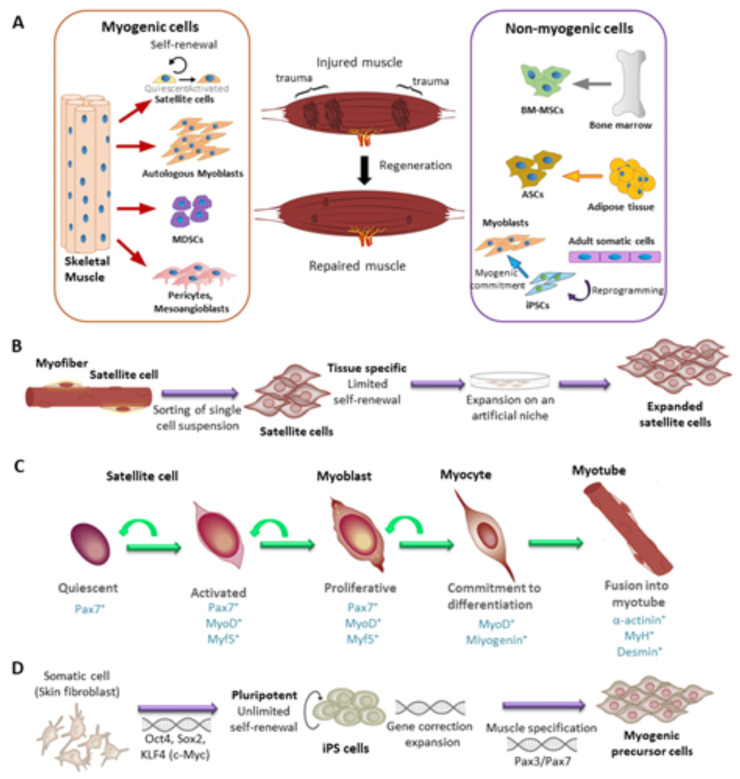
(**A**) Schematic of candidate cells of myogenic or non-myogenic origin, including satellite cells (SCs), myoblasts, muscle-derived stem cells, pericytes, mesoangioblasts, and mesenchymal stromal cells (MSCs) from bone marrow or adipose tissue, and induced pluripotent stem cells (iPSCs). Reproduced from [15] Copyright 2019 open access by Creative Commons Attribution 4.0 International License. (**B**) Schematic of SC isolation and in vitro expansion on artificial niches. (**C**) Differentiation steps of adult myogenesis showing the myotube formation from SCs. After muscle injury or within normal muscle turnover, quiescent SCs are activated. (**D**) Schematic of somatic cell reprogramming to obtain iPSCs and subsequent formation of skeletal myogenic progenitors. (**B**–**D**) reproduced from [17] Copyright 2015 with permission from Elsevier.

Cell therapy is the most commonly utilized approach to improve skeletal muscle regeneration. To obtain a successful treatment, the application of the appropriate cell source is of great importance. To date, various cell candidates including SCs, myoblasts, muscle-derived stem cells (MDSCs), pericytes and mesoangioblasts, bone marrow-derived mesenchymal stromal cells (BM-MSCs), or adipose tissue-derived stem cells (ADSCs), and induced pluripotent stem cells (iPSCs) have been reported for skeletal muscle regeneration (Figure 2A) and their transplantation for the treatment of muscle injury has been investigated [15,16].

For an efficient cell-based treatment, it is necessary to obtain sufficient cell numbers for the application. Furthermore, the applied cells should prevent rejection reactions to eliminate the need for long-term immunosuppressants, preserve myogenic potential during in vitro culture, maintain viability, improve the muscle function permanently after the transplantation process, interact and integrate with the host tissue both structurally and functionally, and replenish and renew stem cell niches located in the tissue.

### 3.1. Satellite Cells (SCs) and Myoblasts

SCs are the muscle stem cells responsible for skeletal muscle growth and regeneration (Figure 2B). They are located between the sarcolemma and basal lamina of myofibers and are also found close to blood capillaries [18]. SCs are found in higher density near neuromuscular and myotendinous junctions. They express the transcription factors Pax7 and Pax3, as well as myogenic factor 5 (Myf5) [19,20,21]. Pax7 is the most important marker for the maintenance of SC function and can be expressed particularly in quiescent and proliferating SCs [17,22]. In early postnatal muscles, SCs account for approximately 30% of the sublaminal nuclei on myofibers, whereas this number decreases to only 2–7% in healthy adults [23,24]. During muscle growth, in the juvenile growth phase, SCs can proliferate and provide nuclei for the growing myofibres [24]. In adults, SCs are generally mitotically quiescent. Once activated, they migrate to the injury site and differentiate into skeletal myoblasts to regulate muscle regeneration (Figure 2C) [25].

SC myogenesis is activated by proximal signals induced by muscle injury or disease [25]. Upon activation, SCs move out of the basal lamina, upregulate the expression of MyoD, Pax7, and Myf5, and then undergo a cell cycle to generate myoblasts for muscle regeneration. After several cycles of cell division, myoblasts exit this cycle and fuse directly with the existing injured myofibers, or they fuse to generate multi-nucleated myotubes following the increased expression of myogenin (Myf4) in the terminal differentiation process. These myotubes can form mature myofibers [16,19,26]. Due to their self-renewal ability, a small number of SCs remain in a quiescent state to replenish the stem cell pool. Therefore, the number of satellite cells remains constant throughout life after repeated cycles of injury and regeneration [24,27,28].

SCs are sensitive to their surrounding niche, which consists of several cell types, including fibroblasts, endothelial cells, immune cells, and inflammatory cells. These cells have been shown to mediate the proliferation and differentiation of SCs [29,30]. In particular, it has been reported that fibroblasts significantly modulate the SC niche and that after injury, the absence of fibroblasts can lead to the formation of smaller regenerated fibers [29]. In a study, Murphy and coworkers showed that the interaction of fibroblasts and SCs can improve muscle regeneration and the fibroblasts regulate SC expansion during repair [31]. Czajka et al. successfully used human endothelial and fibroblast cells to enhance vascularization and SC activation for the treatment of skeletal muscle injury [32].

Since SCs play a crucial role in muscle regeneration, numerous studies have been conducted to investigate SC transplantation into injured muscles. Collin et al. reported that as few as seven SCs associated with one microfiber were able to generate over 100 new myofibers and maintain the stem cell pool after transplantation in mice via the self-renewal capacity of SCs [18]. In this study, SCs derived from different hindlimb muscles resulted in different behavioral characteristics after transplantation into the same host environment. Montarras et al. used a Pax3^GFP/+^ mouse line for the direct isolation of Pax3-GFP expressing SCs by flow cytometry [33]. After transplantation of these cells into dystrophin-null mice, they induced fiber repair and contributed to the SC pool. Importantly, the in vitro expansion of SCs before implantation decreased their regenerative capacity. Sacco et al. also demonstrated the ability of single isolated SCs to self-renew after in vivo transplantation and also showed remarkable cell proliferation and differentiation capacity to induce myofiber formation [34]. The potency of SCs was also demonstrated by the transplantation of only 900 FACS-isolated SCs, which resulted in the measurable improvement of muscle function in a dystrophic mouse model [35].

SC-derived myoblasts involved in skeletal muscle generation have been extensively studied in cell-based therapies for skeletal muscle regeneration. Initial trials confirmed that myoblast transplantation can improve muscle regeneration in mice [36,37]. Partridge and coworkers demonstrated that the intramuscular injection of normal myoblasts into dystrophin-deficient mdx mice resulted in fusion with host muscle fibers and generated myofibers, which expressed dystrophin [38]. Several other studies performed by transplanting myoblast into mdx mice confirmed improved muscle regeneration [39,40,41].

In addition, the use of tissue-engineered scaffolds for the delivery of SCs was investigated. For instance, Rossi and coworkers embedded SCs in in situ photo-cross-linkable hyaluronan-based hydrogels [42]. The transplantation of hydrogels containing freshly isolated SCs into a mouse model enhanced the number of new myofibers and functional recovery compared with hydrogels containing muscle progenitor cells or hydrogels alone. Recently, Prüller and coworkers encapsulated different sources of murine and human myogenic cells into collagen I, fibrin, or PEG-fibrinogen gels [43]. Mouse skeletal myoblast cell line C2C12, human immortalized C25Cl48 myoblasts, primary in vitro expanded SC-derived myoblasts from EDL muscle, and freshly isolated murine soleus myofibres were used. C2C12 myoblasts in collagen I and fibrin hydrogel formed mainly large multinucleated myotubes. Nevertheless, SCs in collagen I and fibrin hydrogels formed aligned multinucleated, and contractile myotubes. Although the muscle niche in vivo supports the muscle regeneration after the transplantation of freshly isolated muscle stem cells, the cultivation of these cells on rigid tissue culture plates in vitro rapidly diminishes their regenerative potential. Gilbert et al. demonstrated that the self-renewal capacity of these cells was maintained when they were cultured on soft substrates (elasticity of 12 kPa) [44].

Although the transplantation of SCs could be an efficient therapeutic strategy, the heterogeneity of the SC population and the difficulties in isolation remain as challenges [3]. SCs from different as well as the same muscle show variations in their gene expression, proliferation rate, myogenic differentiation capacity, and self-renewal ability [20,21]. For instance, it has been reported that the masseter muscle has poor regenerative capacity compared with limb muscle [45]. In addition, the expansion of freshly isolated SCs in vitro severely decreases their myogenic potential in vivo, leading to difficulties in obtaining a favorable amount of cells to induce regeneration [46]. For example, it has been confirmed that even a 3-day culture period significantly reduces the proliferative and myogenic potential of isolated SCs [33,47].

In early clinical trials, the myoblast implantation failed due to low cell survival and migration as well as immunological rejection [48,49,50]. To date, various strategies have been used to address these drawbacks [51]. For instance, Mooney and coworkers seeded enriched populations of myoblasts into alginate hydrogels, which allowed the sufficient regeneration of muscle tissue. The long-term survival of myoblasts was also significantly affected by the composition and architecture of scaffolds as well as growth factor delivery [52].

To date, various myoblast-incorporated tissue-engineered scaffolds have been developed for the treatment of VML due to injury, tumor excision, or degenerative diseases. Machingal et al. designed a porcine bladder acellular matrix scaffold seeded with rat myoblasts [53]. They mechanically preconditioned these scaffolds in a bioreactor providing cyclic unidirectional stretch and relaxation for 1 week and implanted them in nude mice at the site of a VML injury in the lattissimus dorsi muscle. After 2 months of implantation, significant functional recovery was achieved with myoblast-seeded constructs. Subsequently, this group developed three different constructs consisting of cells resembling myoblasts, myotubes, or a combination of myoblasts and myotubes to analyze the influence of cell composition of the porcine bladder matrix scaffold on functional recovery [54]. One construct was generated by the short-term cellular proliferation of rat muscle-derived cells without bioreactor preconditioning to primarily generate myoblasts. The second type of construct was obtained via long-term cell differentiation and preconditioning in a bioreactor to primarily create myotubes. The third type of construct was generated by a similar treatment as the second type of construct, but with a second application of rat muscle-derived cells during bioreactor preconditioning, which was intended to primarily create myotubes and myoblasts, simulating exercise. After the implantation of these constructs into the VML injury model in mice, the first and second type constructs displayed similar functional recovery, while the third type of constructs promoted an accelerated and prolonged functional recovery with twice the magnitude of functional recovery as the other constructs. Furthermore, they implanted the second type of constructs into a VML defect model in Lewis rat tibialis anterior muscle and detected a 2.3-fold higher functional recovery compared to porcine bladder acellular matrix scaffolds without cells [55].

### 3.2. Mesenchymal Stromal Cells (MSCs)

Mesenchymal stromal cells (MSCs) are multipotent stem cells capable of producing cells of mesodermal origin, such as osteoblasts, adipocytes, chondrocytes [17,19]. Several studies also demonstrated the contribution of MSCs in skeletal muscle regeneration [56,57,58]. However, the efficacies reported for myogenic differentiations of human MSCs are a matter of debate. MSCs can also participate in the formation of ectodermal and endodermal tissues in addition to skeletal muscle and play a crucial role in angiogenesis and regeneration of peripheral nerves, which are also important for skeletal muscle repair [59]. They can be easily isolated from various tissues [60], such as adipose tissue, placenta, bone marrow, periosteum [61], and Wharton’s jelly [62]. Importantly, MSCs have immune evasive characteristics. The culture-expanded MSCs typically express low levels of MHC I and no MHC II [16,63]. However, MSCs exposed to IFN-γ or differentiated into mature cell types can express significantly more MHC class I and MHC class II [64]. There is considerable interest in the application of MSCs for skeletal muscle repair for both systemic and local administration.

Bone marrow-derived MSCs (BM-MSCs) represent a valuable multipotent, non-hematopoietic adult stem cell type, which can be also used for skeletal muscle repair due to their myogenic potential [65]. They have the potential to differentiate into myoblasts using myogenic media, co-culture with myoblasts, or biophysical stimuli [16]. Previous studies have shown that BM-MSCs contribute to myogenesis after local (e.g., intramuscular injection) and systemic administration (e.g., intravenous or intra-arterial injection) [66,67,68,69]. For example, Andrade et al. injected BM-MSCs directly into the damaged rat muscle, which increased maximal skeletal muscle contraction, the cross-sectional area of muscle fibers, and the number of mature muscle fibers compared with the non-treated group 28 days post-injury [70]. Helal et al. reported that the intramuscular injection of BM-MSCs into the muscle injury site reduced local immune responses and accelerated muscle fiber regeneration by suppressing inflammatory cytokines and upregulating the anti-inflammatory cytokine IL-10 [71]. In addition, improved angiogenesis was detected and BM-MSCs prevented fibrosis by downregulating transforming growth factor-beta 1 (TGF-β1) and reducing collagen production at the site of muscle injury. It is important to note that a logarithmic dose–response relationship was shown between the number of transplanted BM-MSCs and the rate of muscle recovery following intramuscular BM-MSCs injection in severe skeletal muscle injury in rats [72]. Maeda and coworkers demonstrated facilitated muscle recovery after BM-MSC transplantation into the peritoneal cavity of DMD model mice with severe muscle degeneration [73]. They also concluded that CXCL12 is crucial for muscle regeneration. A study conducted by Sassoli and coworkers showed that platelet-rich plasma (PRP) induced the expression of MyoD, myogenin, α-sarcomeric actin, and MMP-2 in myoblasts and the activation of SCs [74]. The combination of PRP and BM-MSC was found to be more effective compared with PRP alone. Moreover, they reported that BM-MSCs directed the response of myoblasts by paracrine activation of AKT signaling. Natsu and coworkers isolated BM-MSCs from enhanced green fluorescent protein (eGFP) transgenic SD rats, embedded these cells in a fibrin scaffold, and transplanted them into the injured muscle region of SD rats [75]. Transplanted BM-MSCs improved the maturation of myofibers as well as functional recovery one month after transplantation. While they differentiated into muscle progenitor cells, transplanted BM-MSCs failed to differentiate into or fuse to myofibers, suggesting that BM-MSCs may play an indirect role in skeletal muscle regeneration via inflammatory cells. Merritt et al. transplanted a muscle-derived decellularized ECM scaffold into a full-thickness defect in the lateral gastrocnemius of rats and after seven days injected BM-MSCs into the implanted ECM scaffold [76]. The combination of the ECM scaffold and BM-MSCs revealed increased vascularization and skeletal muscle regeneration, resulting in improved structural and functional recovery compared to decellularized ECM alone. In another study, the co-culture of BM-MSCs and MSCs of skeletal muscle-origin resulted in the fusion of both co-cultured cell types after 24 h, and cells with a myotube-like shape were detected after 5 days of cultivation [77].

Adipose-derived stem cells (ADSCs) represent an abundant, adult, and easily expandable multipotent MSC source and show similar myogenic potential to BM-MSCs. They can be easily isolated from adipose tissue in high yield by a simple liposuction procedure and can be differentiated into myoblasts using a myogenic medium containing myogenesis-inducing factors co-culture with myoblasts, biophysical stimuli, or the genetic modification of ADSCs. Notably, they exhibit similar myogenesis potential to SCs when Pax7, Myf5, and MyoD are expressed [16,78]. Di Rocco and coworkers demonstrated that a small number of freshly harvested ADSCs spontaneously differentiate into myogenic cells, indicating their intrinsic myogenic potential [79]. The ADSCs fused into myotubes only when cultured in direct contact with myoblasts. They observed that the injection of freshly isolated ADSCs resulted in fusion with muscle fibers to achieve muscle recovery in mdx mice with ischemic skeletal muscle and significantly restored dystrophin expression. Similarly, the injection of ADSCs into the site of injury was shown to control inflammation, improve angiogenesis, enhance muscle repair, and restore dystrophin expression in mouse models of muscular dystrophy [80]. In another study, Kesireddy used a mouse VML injury model to compare the skeletal muscle regeneration potential of ADSCs and muscle-derived progenitor cells (MDCs) seeded onto decellularized porcine urinary bladder matrix scaffolds [81]. Based on histological analysis, both constructs seeded with ADSCs or MDCs displayed similar regenerative capacity with signs of neotissue formation such as cell fusion, fiber formation, and scaffold remodeling. However, immunohistochemical analyses of the lentivirus-Cherry-labeled donor cells demonstrated that the contribution of ADSCs to the formation of new hybrid myofibers was fairly low compared with the donor MDCs. Nevertheless, ADSCs appeared to be involved in vascularization, which may compensate for the lower efficiency in myofiber formation.

Meligy and co-workers investigated the in vitro isolation efficiency and differentiation capacity of MSCs from adipose tissue (ADSCs), bone marrow (BM-MSCs), and skeletal muscle tissue (MC-MSCs) [82]. Following isolation, MSCs were treated with 5-azacytidine to induce myogenic differentiation. Based on immunocytochemical analyses, ADSCs exhibited the highest proliferation rate and BM-MSCs the lowest. In addition, the differentiation potential of MSCs was assessed by detecting myogenin expression. MC-MSCs had the highest myogenin expression (93%), followed by BM-MSCs (83.3%) and AD-MSCs (77%). The highest percentage of CD90- and CD44-expressing cells was in AD-MSCs and the lowest in MC-MSCs. Even though MC-MSCs showed the highest myogenic differentiation potential, AD-MSCs are the most easily accessible cells and revealed the highest growth as well as the highest percentage of stem cell marker expression.

### 3.3. Muscle Derived Stem Cells (MDSCs)

MDSCs are a distinct stem cell population found in adult skeletal muscle [17]. These cells were isolated based on their adhesion to collagen-coated flasks by the preplating technique. In particular, early adhering and late adhering cells express satellite and stem cell markers, respectively [28,67,83]. Accordingly, they are positive for myogenic markers such as desmin, myogenin, and MyoD and stem cell markers such as Flk-1 and Sca-1, whereas they are negative for the hematopoietic stem cell markers CD45, CD43, and c-kit [83,84,85]. Importantly, they can differentiate into myotubes, self-renew, and be expanded up to 30 passages while maintaining their myogenic potential [28,83,84]. Moreover, they also have a high proliferation and differentiation potential, show high survival rates under hypoxic conditions, and resistance to oxidative stress [86,87,88]. MDSCs are a precursor of SCs with the ability to differentiate not only into myogenic lineage but also other mesodermal lineages [88] and differ from SCs [15]. MDSCs could be particularly beneficial when the injury affects the myotendinous (muscle–tendon interface) junction and the muscle-associated tendon [15].

The transplantation of MDSCs into the dystrophic mice model promoted muscle regeneration and created hybrid fibers expressing dystrophin after their local or systemic injection [88,89]. A study by Qu-Petersen and coworkers showed that the transplantation of MDSCs resulted in 10 times more dystrophin^+^ myofibers compared with the transplantation of SCs [90]. Rouger et al. demonstrated that the intra-arterial injection of MDSCs in immunosuppressed dystrophic dogs resulted in improved clinical benefits through long-term dystrophin expression and enhanced myofiber regeneration, the replenishment of SCs, and increased walking ability [91]. Lorant et al. reported long-term proliferative capability and myogenesis in vitro and also improved skeletal muscle regeneration following intramuscular injection into immunodeficient mice [92]. Recently, Matthias and coworkers transplanted MDSCs using an in situ fibrin gel cast into a muscle loss injury model. It was found that transplanted MDSCs in fibrin gel were able to engraft, form new myofibers, increase muscle mass, reduce fibrotic tissue, promote new vessel formation, and restore muscle SCs [86].

In a clinical study, a DMD patient received a high-density injection of normal muscle-precursor cells cultivated in vitro and was immunosuppressed with tacrolimus [93]. The expression of donor-derived dystrophin was still detected 18 months post-transplantation. Furthermore, in a very recent study, Klimczak et al. simultaneously co-transplanted BM-MSCs and skeletal muscle-derived stem/progenitor cells in three DMD patients, which resulted in increased motor unit parameters [94]. Furthermore, decreased creatine kinase levels and a normalized profile of pro-inflammatory cytokines were detected. This indicates that BM-MSCs might support the regenerative potential of skeletal muscle-derived stem/progenitor cells due to their trophic, paracrine, and immunomodulatory activity. Thus, both cell types could facilitate skeletal muscle recovery by the fusion with degenerating skeletal muscle fibers in vivo.

### 3.4. Pericytes

Pericytes are perivascular stem cells located in capillary and microvascular walls. These cells are found underneath the microvascular basal lamina and make contact with the capillary endothelial cells to modulate microcirculation [16,95]. Pericytes have been isolated from various tissues, such as adipose tissue, skeletal muscle, and the pancreas, and can differentiate into adipogenic, chondrogenic, and myogenic cells [19,96]. Pericytes do not usually express Pax7 or MyoD and express myogenic markers only post-differentiation [19,97,98]. Type 1 (nestin^−^ NG2^+^) and type 2 (nestin^+^ NG2^+^) pericytes have been identified as two major subpopulations. Type 1 pericytes are involved in fat accumulation and type 2 pericytes are known to support new muscle formation [97,99].

Dellavalle and coworkers demonstrated that pericytes contribute to skeletal muscle fiber development and the SC pool during postnatal growth [98] and participate in tissue regeneration. The myogenic potential of pericytes was shown by the formation of a high number of differentiated muscle fibers expressing human dystrophin after injection into immunodeficient SCID–mdx dystrophic mice [96]. They also proved that the pericytes isolated from normal and dystrophic human skeletal muscle can be expanded to obtain sufficient cell numbers for the treatment of a pediatric patient. These cells can be transduced with viral vectors to express dystrophin and differentiated into skeletal muscles for DMD therapy. A study by Fuoco et al. presented an approach to rejuvenate adult pericytes using a PEG-based hydrogel scaffold [100]. Pericytes were isolated from young and adult pigs to investigate the effect of aging on muscle regeneration. The morphology and colony-forming potential of adult pericytes were similar to those of young pericytes. However, their myogenic potential was reduced with aging due to their decreased ability to form myotubes and capillary-like structures. Remarkably, the culture of adult pericytes on a PEG-based hydrogel significantly enhanced their myogenic and angiogenic activity in vitro and in vivo. Therefore, it was speculated that PEG-based 3D hydrogels mimic the stiffness and mechanical cues of the muscle ECM and lead to the rejuvenation of myogenic potential.

### 3.5. Induced Pluripotent Stem Cells (iPSCs)

iPSCs can be generated in vitro by introducing reprogramming factors, known as Yamanaka factors, into somatic cells. They possess unlimited self-renewal capacity in culture and can be differentiated into almost all cell types [30,101]. Thus, iPSCs offer an attractive option for myogenic regeneration due to their ability to differentiate into myogenic cells [19,102]. Importantly, the use of patient-specific iPSCs overcomes major limitations of human embryonic stem cells (ESCs), including ethical concerns and rejection reactions [17]. iPSCs can be generated, e.g., from a patient´s fibroblasts obtained by a skin biopsy or from renal epithelial cells obtained non-invasively from urine [103]. These iPSCs have an unlimited proliferative capacity and enable the in vitro expansion of a patient´s autologous cells in large quantities, which can then be differentiated into autologous myocytes (Figure 2D) [16,17]. The differentiation of iPSCs into myogenic cells can be performed by the overexpression of Pax7 or MyoD [104]. Alternatively, small molecules can be used to perform transgene-free differentiation. However, it is difficult to generate a pure, expandable myogenic population using this method [104]. In particular, the use of genome-integrating vectors to perform reprogramming can provoke genetic instability and abnormalities and promote tumor formation. Thus, researchers are focusing on the generation of iPSCs as well as myogenic differentiation without genomic modifications [16,105,106].

To date, several studies have been performed for the myogenic differentiation of iPSCs to generate functional skeletal muscles in vitro as well as to fuse these cells with existing myofibers after in vivo transplantation [107,108,109,110,111,112]. Darabi and coworkers generated iPSCs from human fibroblasts and induced Pax7 for their differentiation into myogenic progenitors with the ability to expand in vitro [113]. The transplantation of these myogenic progenitors into dystrophic mice enhanced contractility and resulted in the formation of dystrophin-positive myofibers. Large quantities of myogenic progenitors were obtained by the conditional expression of Pax7 in human ESCs and iPSCs, and an improved muscle fiber formation was achieved following their engraftment in immunodeficient dystrophic mice [114]. Mondragon-Gonzalez et al. generated iPSCs from fibroblasts of patients with myotonic dystrophy 1 (DM1) using Sendai virus transduction. These iPSCs were then differentiated into myogenic progenitors using a Pax7-containing lentivirus vector and differentiated into myotubes.

Shoji et al. presented a reproducible myogenic differentiation protocol for iPSCs using a tetracycline-inducible myogenic differentiation 1 (MYOD1) piggyBac (PB) vector [115]. Their protocol resulted in a homogenous skeletal muscle cell population with high differentiation efficiency (70–90%). Recently, a serum-free myogenic differentiation protocol was developed by Chal and coworkers and muscle fibers and satellite-like cells were efficiently produced by realizing key embryonic signaling events including the alteration of both Wnt and bone morphogenetic protein (BMP) pathway signaling [108]. Hosoyama and coworkers used free-floating spherical culture (EZ spheres) in a defined culture medium to obtain myogenic progenitors from human ESCs and iPSCs [116]. Following 6 weeks of culture in a medium containing high concentrations of fibroblast growth factor-2 (FGF-2) and epidermal growth factor (EGF), myogenic progenitors from human iPSCs were observed, and after a further 2 weeks of terminal differentiation, multinucleated myotubes expressing Pax7, MyoD, MHC, and myogenin were found. Similarly, Jiwlawat et al. studied the time course for muscle differentiation and sarcomere formation in EZ sphere-derived myogenic progenitors [117]. They reported that a differentiation period of at least 6 weeks was required for the differentiation of iPSCs into myogenic progenitors and the generation of mature skeletal myotubes with organized sarcomeres. Borchin and coworkers successfully used a small-molecule GSK3β inhibitor (CHIR99021) to activate the Wnt signaling pathway for myogenic cell production [118]. They utilized fluorescence-activated cell sorting (FACS), which allowed the purification of the resulting PAX3^+^/PAX7^+^ skeletal muscle precursors that were able to differentiate into mature myocytes. Subsequently, van der Wal and coworkers modified Borchin´s differentiation protocol and used a higher concentration of CHIR99021 (3.5 μM) for a longer duration (5–6 days), resulting in a higher yield of Pax7^+^ cells [119]. To purify Pax7^+^ cells, FACS was performed using C-Met^+^/Hnk1^−^ cells. The obtained cells differentiated into multinucleated myotubes with a high fusion index. They also demonstrated their efficient contribution to myofiber formation after intramuscular engraftment into pre-injured muscles of mice in vivo [102].

Goudenege et al. cultivated dystrophic human iPSCs first in a myogenic medium and the cells were then transfected with an adenoviral vector for the expression of MyoD [109]. This two-step protocol resulted in the formation of multinucleated myotubes in vitro. The transplantation into the muscle of Rag/*mdx* mice led to the regeneration of muscle by fusion with existing muscle fibers. Recently, Baci et al. established a transgene-free protocol for the myogenic differentiation of iPSCs generated from muscular pericytes or fibroblasts using skeletal muscle-derived extracellular vesicles (EVs) cargo and GSK-3 inhibitor [120]. After 30 days, increased muscle differentiation was detected in vitro by the enhanced expression of myogenic markers. Subsequently, differentiated iPSCs were injected into the muscle of mice, and these cells integrated into the regenerating host myofibers. Moreover, the EVs applied were efficient as biological “shuttles” for delivering bioactive molecules to induce the differentiation of iPSCs.

## 4. Biomaterials for the Transplantation of Striated Muscle Cells

In current clinical treatments, the transplantation of allogeneic muscle cells is significantly limited due to rejection reactions, donor site morbidity, and insufficient donor cells [121]. In addition, the delivery and retention of cells in muscle are inefficient, hindering the sustained regeneration required for sufficient functional improvement [122,123]. Alternative strategies are therefore required. Biomaterials are powerful tools to support the cellular microenvironment as well as to provide sufficient contractile strength to match native skeletal muscle [8,123,124,125].

Various biomaterial-based strategies with different physical and chemical behaviors have been investigated for the treatment of muscle injuries [8]. Hydrogels are 3D crosslinked hydrophilic polymeric matrices with a high water content that closely mimic the native aqueous environment of the body [125,126]. They have structural similarities to the natural ECM and can be administered with less invasive strategies [122]. Injectable hydrogels can be administered directly to the damaged site for the controlled release of therapeutics. Several biodegradable hydrogels, such as collagen [127,128], hyaluronic acid [129], alginate [130,131,132], fibrin [133,134], and synthetic hydrogels have been studied as injectable forms. On the other hand, self-healing, injectable hydrogel-based constructs are more effective as cell carriers due to their homogeneous loading behavior and cause less discomfort to the patient [135].

In this section, natural, synthetic, and composite hydrogel-based biomaterials for the transplantation of striated muscle cells are discussed.

### 4.1. Natural Hydrogels

Hydrogels based on natural elements are ideal candidates as they elicit a limited inflammatory response, have structurally similar elements to the body and they are highly effective in triggering the skeletal muscle regeneration process [136,137]. Recent strategies have focused on naturally derived hydrogel systems to supply essential biochemical signals required for cell adhesion, proliferation, and myogenic differentiation [125]. Especially, the integration of ECM components into the composition of biomaterials holds great promise for use in muscle regeneration [8]. Collagen, gelatin, fibrin, matrigel, keratin, hyaluronic acid, silk, and alginate-based hydrogels are some of the most investigated materials for skeletal muscle tissue engineering [126,127,138].

Collagen, the major structural component of ECM, has been used extensively in muscle tissue engineering, where its biocompatibility and resorption make it an attractive choice [138,139,140,141]. Li and coworkers developed a myoblast-seeded vascularized collagen hydrogel scaffold and implanted it into a VML model consisting of a full-thickness, single muscle defect in the rat biceps femoris muscle to show the importance of vascularization in muscle recovery [142]. However, vascularized scaffolds with or without myoblasts resulted in the formation of fibrotic tissue with adipose infiltration, low muscle regeneration, and poor maintenance of tissue volume at the injury site. These results suggest that besides vascularization, other factors also appear to be crucial to the fate of regeneration. Basurto et al. produced aligned and electrically conductive 3D collagen scaffolds [5]. Therefore, conductive polypyrrole (PPy) microparticles were incorporated into a suspension of type I collagen and chondroitin sulfate. Thereby, the conductivity was increased five-fold compared to non-PPy containing collagen scaffolds. Furthermore, myotube formation and maturation were increased. The aligned microstructure of the scaffold guided the directed growth and organization of myoblasts.

**Figure 3:**
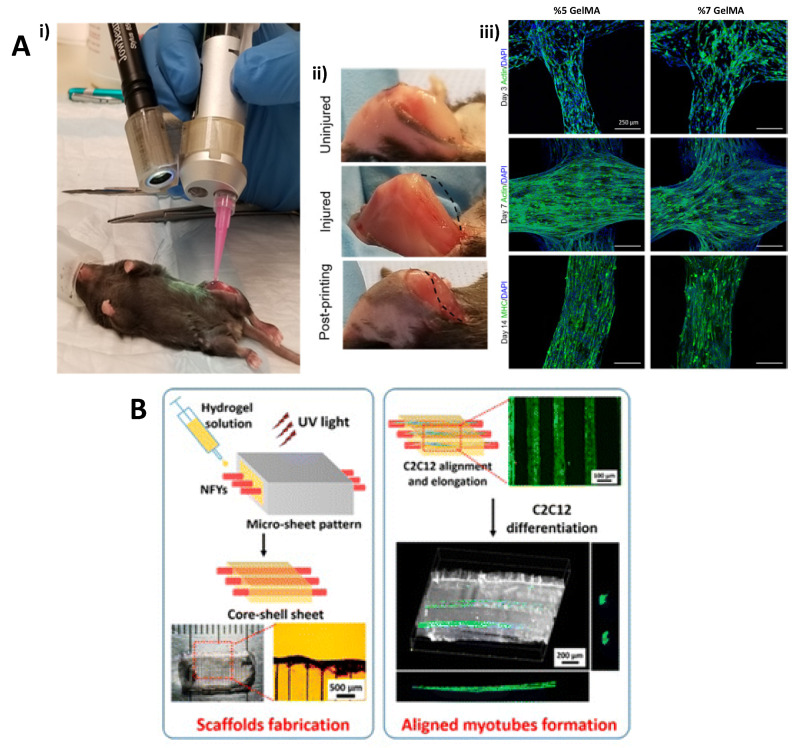
(**A**) Hand-held printed gelatine-methacryloyl (GelMA) hydrogels. (**i**) In vivo application of GelMA hydrogel scaffolds, (**ii**) before VML surgery, post-VML surgery, post in situ printing of GelMA hydrogel, (**iii**) F-actin/DAPI staining of encapsulated cells in GelMA construct. Reproduced with permission from [14] Copyright (2020) American Chemical Society. (**B**) Preparation of composite hydrogel scaffolds by incorporating the aligned nanofibers yarns and formation of aligned myotubes. Reproduced with permission from [145]. Copyright (2015) American Chemical Society.

Gelatin is a denatured low-cost form of collagen that can be cross-linked into tunable constructs with elastic moduli similar to native muscle. Gelatin hydrogels crosslinked with microbial transglutaminase (MTG), an FDA-approved enzyme, improved the long-term culture and maturation of myotubes [143]. Bettadapur et al. were able to maintain differentiated skeletal myotubes from C2C12 mouse skeletal myoblasts aligned for three weeks [144]. Compared to polydimethylsiloxane (PDMS) microcontact printed scaffolds with fibronectin, cell adhesion on gelatin hydrogel constructs was significantly higher. In another study, Russell et al. printed gelatine–methacryloyl (GelMA) hydrogels directly into the site of injury of skeletal muscle tissue using a hand-held, partially automated-bioprinter and GelMA hydrogels supported the formation of multinucleated myotubes (Figure 3A) [14].

Collagen type I hydrogels can mimic the native environment of skeletal muscle tissue, but their relatively high stiffness hinders long-term muscle culture, differentiation, and contractile force generation [146]. In contrast, fibrin hydrogels provide a temporary scaffold for cells that can be remodeled and replaced by the newly synthesized ECM. Fibrin is the main component of blood clots, which is generated after tissue injuries. The fibrin sealant tissue adhesive is an FDA-approved material. In addition, compared to collagen, fibrin is a low-cost material whose mechanical behavior and degradation profile can be adjusted by using different fibrinogen concentrations or crosslinker [122,147].

The co-culture of muscle and endothelial cells in fibrin-based hydrogels enabled the formation of aligned myofibers and interconnected endothelial networks [148]. Marcinczyk et al. investigated the material properties of laminin-111-incorporated fibrin hydrogels, capability for myogenesis, and response to electromechanical stimulation [147]. The release of LM-111 from fibrin-based hydrogels on the muscle defect area was found to be an effective strategy to promote myoblast proliferation and pro-regenerative growth factor secretion. The combined application of electromechanical stimulation significantly increased the production of VEGF and IGF-1 from myoblast-seeded fibrin LM-111 hydrogels. Gilbert-Honick and coworkers evaluated murine myoblast-seeded electrospun fibrin hydrogel scaffolds in a murine VML injury model with defects to the tibialis anterior muscle [10]. Myoblast-incorporated scaffolds revealed significantly improved muscle repair and a high number of myofiber and vascular densities, whereas acellular scaffolds resulted in impaired regeneration. Notably, myoblast-seeded scaffolds enabled the complete regeneration of the structure and function of volumetric muscle defect.

Alginate is a natural polysaccharide, which is biocompatible, biodegradable, non-toxic, non-immunogenic, and easy to process. It is also inexpensive compared to many other biomaterials [149]. However, alginate-based constructs show a weak cell adhesion, which may cause unfavorable tissue interactions and insufficient regenerative behavior [150]. The functionalization of alginate with cell adhesion-promoting ligands, such as arginine–glycine–asparagine RGD can support the interaction of the cells with the alginate matrix [151]. The delivery of growth factors from RGD-conjugated alginate scaffolds was shown to promote the survival and outward migration of primary myoblasts [152]. In another study, alginate-based hydrogels with a stiffness of 13–45 kPa were found to improve myoblast proliferation and differentiation [153]. Yeo and colleagues combined a process involving electrospinning and myoblast printing to achieve a hierarchical structure [154]. Thereby, an alginate bioink containing polyethylene oxide (PEO) and C2C12 myoblasts was printed on polycaprolactone (PCL) struts, micro/nanofibers. The presence of micro/nanofibers improved the proliferation of myoblasts, alignment, and the formation of myotubes.

### 4.2. Synthetic Hydrogels

Synthetic hydrogels have been a less popular choice for skeletal muscle tissue engineering compared to natural polymer-based hydrogels. However, synthetic polymer-based hydrogels can be easily engineered to enable the controlled release of growth factors to promote muscle regeneration. The major limitations are typically weaker cell adhesion compared to hydrogels based on natural polymers and the risk of the stimulation of a foreign body reaction by the polymer or its degradation products [2,124]. Synthetic polymers such as poly-L-lactic acid (PLLA) [155,156], poly(lactic-co-glycolic acid) (PLGA) [156], PCL [157,158,159,160], poly(ethylene glycol) (PEG) [161,162,163] and their copolymers [164], and various polyurethanes (PU) [165,166,167,168] are preferred for musculoskeletal tissue engineering. Among the synthetic polymers, elastomers, such as polydimethylsiloxane (PDMS), have been extensively used for the 2D fabrication of tissue-engineered muscle thin films due to their excellent biostability and tunable elasticity [169,170]. Ergene et al. engineered a new PU-based biodegradable elastomer to mimic elastic behavior and a native dynamic micro-environment of muscle tissue [167]. Han et al. developed a synthetic PEG-based hydrogel to enable the co-delivery of SCs and promyogenic factors, such as Wnt7a for skeletal muscle regeneration [162]. Mechanical behaviors and the chemical composition of synthetic polymers (e.g., degradation profile, stiffness/ rigidity) can be more precisely tuned compared to natural-based biopolymers, and some synthetic-based biopolymers. They can also be produced to be electrically conductive. Since electroactive polymers, which change their size or shape in response to an electric field, may contribute to an optimal environment for muscle growth, a biocompatible electroactive hydrogel made of PEG diacrylate (PEGDA) and acrylic acid (PEGDA/AA) was developed [171]. Conductive hydrogels were generated by incorporating graphene oxide (GO) [172] or polyaniline (PANi) [173] into the matrix formulation [173,174]. Thereby, hydrophilic, soft, and conductive scaffolds, composite structures, were created, which supported the growth of skeletal muscle. In the following section, the advantages and disadvantages of composite scaffolds for potential application to regenerate skeletal muscle tissue are discussed in detail.

### 4.3. Composite (Hybrid) Hydrogels

The combination of natural and synthetic polymers in composite (hybrid) hydrogels can enable the creation of scaffolds with improved skeletal muscle regeneration [175,176]. Wang et al. designed PEG-co-poly(glycerol sebacate) composite hydrogel scaffolds by incorporating the aligned nanofibers yarns. These yarns with hybrid composition were produced by combining PCL, PANi, and silk fibroin (SF) using the dry–wet electrospinning method to enhance the mechanical stability of scaffolds, cell adhesion, and proliferation behaviors [145] (Figure 3B). The designed platform guided the cellular alignment along the direction of the yarns and provided a suitable 3D environment for skeletal muscle regeneration. In another study, the incorporation of the fibrinogen in PEG composite scaffolds rejuvenated adult skeletal muscle-derived pericytes, improved their myogenic differentiation and angiogenic capacities in vitro and in vivo [100]. Furthermore, by the incorporation of PANi in a PEG-hydrogel and the use of UV-induced photolithography with photomasks, electrically conductive hydrogel micropatterns were created [177]. Thereby, the myogenic differentiation of the C2C12 cells was induced and the alignment of myotubes was improved.

Ku et al. reported that the implementation of GO into the substrate formulation remarkably improved myogenic protein expression, multinucleated myotube formation, and myogenic differentiation [178]. Furthermore, Chaudhuri et al. demonstrated that GO–PCL hybrid scaffolds can enhance myotube formation and maturation [179]. Uehara et al. developed GO-implemented random and aligned electrospun PCL-based nanofibers and investigated the interactions between skeletal muscle cells and the polymer matrix [180]. They reported that GO-implemented aligned nanofibers did not exhibit the same cell orientation as the cells in contact with pure PCL-aligned nanofibers. Carbon nanotubes (CNTs) have been investigated for the formulation of scaffolds for the potential application of skeletal muscle regeneration [181]. Patel et al. built CNT-based hierarchical scaffolds in two different forms, interconnected microporous foams or aligned fiber mats, with tunable physicochemical behavior. Myoblasts on both scaffolds demonstrated similar adhesion and proliferation properties. The scaffold with aligned fibers promoted the fusion of myocytes into myotubes [182]. In contrast, microporous foams failed to promote the fusion of myoblasts into multinucleated myotubes.

## 5. Biomechanical Strategies for the Generation of Skeletal Muscle Tissue

Skeletal muscle tissue engineering has the potential for clinical implementation and enables the accurate modeling of myogenesis and evaluation of pharmacological treatments. However, the clinical applicability of tissue-engineered skeletal muscle is severely hampered by the inadequate generation of contractile forces, myotube alignment, and low packing density and cell survival in large constructs [1,183]. It is important to mimic the natural cell niche in vivo to achieve in vitro myogenesis that resembles native tissue [1,184].

Adult myofibers are terminally differentiated post-mitotic cells. Thus, the remodeling of skeletal muscle is controlled by adaptive mechanisms that induce the accumulation of contractile fiber proteins by either increased protein synthesis or decreased protein degradation. The training and stretching of muscles led to several in vivo adaptations including the regulation of protein synthesis and degradation, DNA and RNA content, and protein accumulation [185]. The mechanical stimulation of skeletal muscle mainly results in remodeling (changes in muscle mass/volume) and alterations in the molecular engine of the muscle fiber, e.g., phenotypical changes in the expression of myosin isoforms [186]. These changes allow the muscle to adapt its contractile properties to the required demands.

Scientists have conducted several studies on mechanical stimulation as it has a significant effect on skeletal muscle cell behavior. Okano et al. incorporated C2C12 cells (mouse skeletal myoblast cell line) into type I collagen gel and formed ring-shaped constructs using agarose gel molds [187]. These rings were subjected to cycling stretching at 60 rpm, resulting in highly dense and oriented muscle tissue that closely resembles native skeletal muscle. Auluck et al. cultivated human craniofacial muscle-derived cells in a sponge made of a 3D collagen network to mimic the environment of the muscle matrix and exposed these constructs to the strain regimes of rapid ramp stretch or cyclical ramp strain [188]. The remodeling of the ECM was detected by increased expression of the mechanoresponsive gene, matrix metalloproteinase-2 (MMP-2), in cultures subjected to continuous versus cyclic strain.

Powell et al. incorporated muscle cells in collagen/matrigel and exposed them to repeated stretch/relaxation cycles using a mechanical cell stimulator (MCS) [185]. After 8 days, a two- to three-fold increase in construct elasticity, 40% in myofiber area, and 12% in mean myofiber diameter was observed. Sakiyama and colleagues showed that the application of a mechanical stimulus can affect not only the growth and differentiation of myoblasts but also the characteristics of muscle fibers [189]. C2C12 myoblasts were mechanically stretched, and then the mRNA expression levels of myosin heavy chain (MHC) isoforms were measured at different time points. The results showed that MHC-2b expression first increased and then decreased after stretching. MHC-2d expression increased over time without stretching but was barely detectable with stretching. In addition, MHC-2a expression level was significantly high in the stretching group. Based on these results, it could be extrapolated that mechanical stimulation affects MHC isoform levels and thereby influences muscle function. In another study, the cyclic mechanical stretch of C2C12 myoblasts increased β_1D_-integrin protein levels, which regulates myogenesis and mechanotransduction, and activated downstream cytoskeletal signaling proteins focal adhesion kinase (FAK) and RhoA [190]. Moon et al. seeded collagen-based acellular tissue scaffolds with primary human muscle precursor cells (MPCs) and subjected them to cyclic strain. These preconditioned constructs in computer-controlled bioreactors produced tetanic and twitch contractile responses after transplantation into mice, but not statically cultured control tissues [191]. Egusa et al. showed the possibility of accelerating the skeletal myogenic differentiation of BM-MSCs with an aligned structure by applying cyclic strain [192]. In this study, mouse BM-MSCs were seeded on fibronectin-coated silicone sheets and subjected to cyclic 10% uniaxial strain. Culturing the cells in a myogenic medium resulted in upregulation of skeletal myogenic marker genes (myogenin, Myf5, and myogenic regulatory factor 4 (MRF4)), but not smooth muscle marker genes (myocardin and α-smooth muscle actin). Moreover, the cells formed multinucleated myotubes in the direction of applied tension within 5 days.

## 6. Scaffold Topographies for Improved Generation of Muscle Tissue

First studied in 1911 [193] and then with advances in contact guidance in 1945 [194], topographical cues were examined for their ability to control cell behavior and orientation. In recent years, the importance of micro/nanostructures has increased, since the cells can respond to topographic properties at the micro/nanometer levels. Thus, the generation of muscle tissue with topographical cues is analyzed using a variety of bioengineering methods. Therefore, different fabrication techniques were applied, such as the passive or active stretching of scaffolds, photolithography, soft lithography, solvent casting, electrospinning, freeze-drying, hot embossing, and the use of electrical fields [124]. The scaffolds in muscle tissue have different geometric structures, including grooves, pillars, pits, wrinkles, as well as fibrous scaffolds combined with micro-/nano-structures [195]. In this section, the role of topographies, such as the use of grooves and fibrous structures, on muscle tissue regeneration are highlighted.

Electrospun fibers can replicate and regenerate tissue environments in a 3D state as they mimic the nanoscale properties of a native ECM. They can also be fabricated both anisotropically and isotropically to control the commitment of muscle cells to a particular lineage [196]. Moreover, a variety of biopolymers and synthetic polymers can be fabricated as micro- and nanoscale fibers [197,198]. The properties of electrospun nanofibers, such as random and aligned structures [199], variable thicknesses, and nanoscale micropores can be assessed in the context of topographical effects. In this regard, the electrospinning technique has been used to fabricate skeletal muscle scaffolds with uniaxially patterned structures that increase the length of myotubes [200]. For instance, the uniaxially patterned electrospun PLLA fibers could enhance the length of myotubes about three-fold in comparison with random fiber mats when C2C12 cells were cultured on aligned PLLA nanofibrous scaffolds [201].

**Figure 4:**
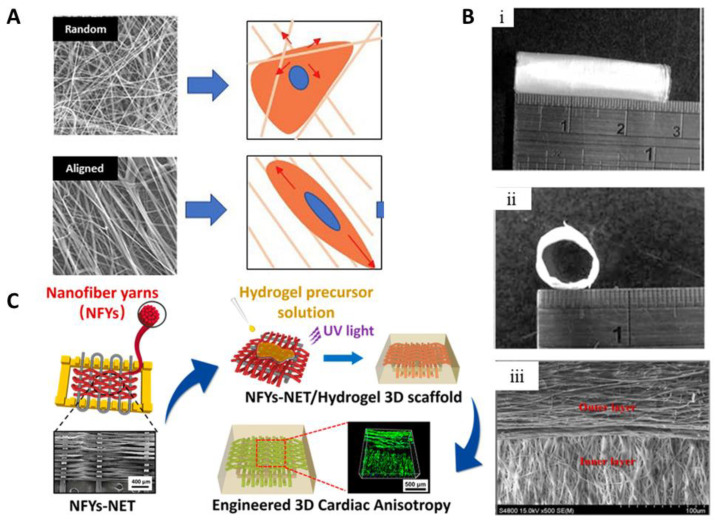
(**A**) Schematic image of fibrinogen substrate electrospun fibers with different topographies, which direct the cell morphology and phenotype. Reprinted from [202]. Copyright 2019, with permission from Elsevier. (**B**) Tubular scaffold structure; (**i**) surface and (**ii**) cross-section images with (**iii**) SEM images of outer and inner layers. Reproduced from [203]. Copyright 2019 with permission from Elsevier. (**C**) Scheme of the nanofiber yarns (NFYs)-network (NET) scaffolds fabricated by a weaving technique and GelMA hydrogel shell layer prepared by UV irradiation. Reproduced with permission from [204]. Copyright 2017 American Chemical Society.

Aligned nanofiber scaffolds can also be used in the development of tissue-engineered vascular grafts (TEVGs) as part of a multi-pronged strategy to enhance elastic fiber formation. Substrate topography and biochemical additives can synergistically increase the elastin fiber formation by cultured cells (umbilical artery smooth muscle cells) on the aligned fiber and thereby improve the quality of TEVGs. Electrospun fibrinogen nanofibers with aligned topography led to increased elastin and phenotypic contractile protein synthesis and the directional orientation of cells as well as newly synthesized ECM fibers (Figure 4A) [202].

Research is conducted on tubular scaffolds to simulate the structures and functions of blood vessels. Here, three layers of tissue, intima, media, and adventitia, with different structures and functions are simultaneously required to repair and regenerate blood vessels. Tsai et al. generated small diameter (<6 mm) nanofibrous tubular PCL grafts with an orthogonal-bilayer structure by electrospinning (Figure 4B) [203]. The inner layer consisted of axial nanofibers, which can provide topographical guidance for ECs, while the outer layer consisted of circumferentially aligned nanofibers to increase mechanical strength and provide topographical guidance for smooth muscle cells. In a recent study, to mimic the structure and function of native blood vessels, Hu et al. fabricated four-layer tubular scaffolds by electrospinning thermoplastic PU (TPU)/PCL/PEG tubular scaffolds, which had an interior layer with highly longitudinal aligned fibers. The middle inner and outer layers were created by sloped and circumferentially aligned fibers and the exterior layer consisted of random fibers. [205]. In addition, tubular scaffolds with completely random fiber layers were produced. The incorporation of PEG improved the hydrophilicity of the scaffolds. The results also showed that the scaffolds with aligned fibers not only improved their mechanical properties but also enhanced the cellular proliferation and viability of HUVECs.

Scaffold design plays an important role in cardiac tissue regeneration to mimic the cardiac anisotropic structure and guide 3D cell alignment. Although several studies have been conducted to control cell alignment and elongation, there are still challenges in the mimicry of the 3D anisotropy of the cardiac muscle. Cardiac muscle PCL patches were produced by using an electrolyte solution as a collector in the electrolyte-assisted electrospinning (ELES) process to hybridize random and aligned nanofibers simultaneously [206]. Thereby, random nanofibers can provide mechanical support to be handled and sutured, while aligned nanofibers improve the alignment of cardiomyocytes and the construction of a 3D anisotropic structure. In further studies, 3D hybrid scaffolds were created with core and shell structures to engineer the 3D cardiac anisotropy and control the orientation of cells [204]. As shown in Figure 4C, 3D hybrid conductive scaffolds were fabricated by encapsulating the nanofiber PCL/SF/CNT yarn network within a GelMA hydrogel shell. Cardiomyocytes were seeded on a yarn network and embedded into GelMA hydrogel containing GFP-expressing ECs. These scaffolds enhanced the maturation and function of cardiomyocytes as well as the alignment and elongation of cardiomyocytes.

Scaffolds with core-shell structures have also been investigated for skeletal muscle tissue engineering. For example, PCL containing multiwalled carbon nanotubes solution and a poly(acrylic acid)/poly(vinyl alcohol) (PAA/PVA) hydrogel have been coaxially electrospun as the core and shell layers, respectively [207]. The multiwalled carbon nanotubes in the PCL core layer act as an internal electrode and increase the conductivity, while the hydrogel shell can actuate. The scaffolds with a hydrogel shell displayed more multinucleated cells with interacting actin filaments compared to the scaffolds without hydrogel.

**Figure 5:**
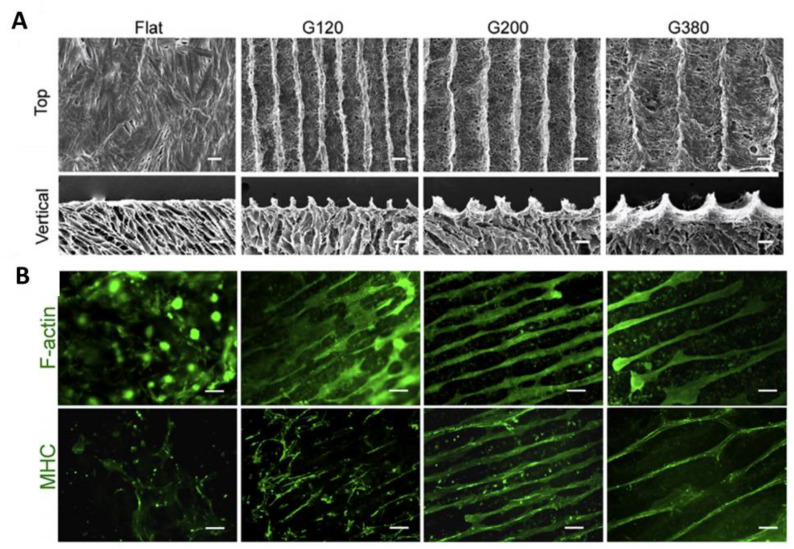
(**A**) SEM images of different microgrooved collagen scaffolds. Top-view and vertical cross-sectional view are shown. Flat collagen scaffold (control), collagen scaffolds with mean microgroove widths of 120 µm (G120), 200 µm (G200), and 380 µm (G380). Scale bar = 100 µm. (**B**) Myotube formation after cultivation of L6 myoblasts for 14 days. F-actin and myosin heavy chain (MHC) staining were performed to visualize myoblasts and myotube formation. Scale bar = 200 µm. Reproduced from [210]. Copyright (2015) with permission from Elsevier.

Micropatterning technology is another promising strategy to generate 3D topographies at micrometer and submicrometer levels. Using this strategy, cell-culture substrates can be designed to engineer aligned myoblasts and myotubes. Hosseini et al. produced micro-grooved GelMA hydrogel substrates using PDMS molds and UV crosslinking [208]. The seeding of murine C2C12 myoblasts within the microchannels led to the myotube alignment. In contrast, no alignment was found on unpatterned hydrogels. Furthermore, the electrical stimulation further improved the myotube alignment and generated myotubes with an increased diameter. This study demonstrated that the behavior and fate of cells, such as cell–cell contact and signaling, differentiation, and elongation, can be altered using micropatterns. Alternatively, photolithography and/or soft lithography are also applied as micropatterning techniques to produce various surface topographies. In one study, the cultivation of human iPSC-derived myogenic progenitors on PDMS-based micropatterned substrate resulted in larger fiber diameters, more myogenin-positive nuclei, and increased nuclear fusion compared to the cells cultivated on non-patterned substrates [209]. Freeze-drying is another technique used for microstructured scaffolds in skeletal muscle tissue engineering. Chen et al. fabricated a 3D porous collagen scaffold with concave microgrooves to mimic the muscle basement membrane [210]. The cultivation of rat L6 skeletal myoblasts on these scaffolds resulted in multilayered muscle bundle tissue in which myoblasts aligned and formed myotubes dependent on the size of the microgrooves and cell density. However, changing the size of the grooves did not affect the expression of myogenic genes. Figure 5 shows scaffolds with mean microgroove widths and the myotube formation after 14 days. The size, ridge, and material type of the grooves can have an impact on cell organization, differentiation, and alignment. Hydrogels with micro-sized structures have a great ability to guide cells into a biomimetic tissue architecture in vitro and can be used as micropatterned substrates in muscle tissue engineering. For instance, two types of micropatterned methacrylated gelatin hydrogel substrateswere produced with the same grooves (100 µm) and distinct ridge patterns (50 and 100 µm) [208]. The results showed that the smaller ridge micropatterns yield more myotubes, which are aligned in the groove direction than the wider ridge micropatterns.

**Figure 6:**
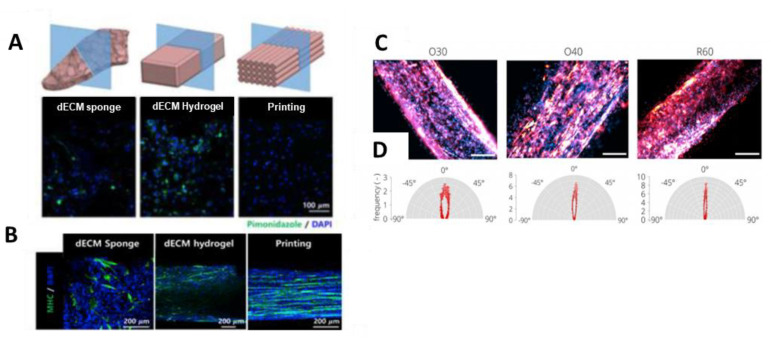
(**A**) Detection of hypoxia in the dECM sponge, dECM hydrogel, and 3D printed muscle constructs with pimonidazole staining and (**B**) immunofluorescence images of human skeletal muscle cells in constructs. Reproduced from [213]. Copyright (2019) with permission Elsevier. (**C**) Fluorescence microscopy images of F-Actin (red)/DAPI (blue) stained C2C12 cells and (**D**) Corresponding frequency of F-Actin filament orientations, O30 (d = 330 µm, p = 30 kPa), O40 (d = 330 µm, p = 40 kPa) and R60 (d = 250 µm, p = 60 kPa), d: nozzle diameter, p: extrusion pressure. Scale bars: 200 µm. Reproduced from [214]. Copyright 2020 open access by Creative Commons Attribution 4.0 International License.

In recent years, efforts to use 3D printing technology to fabricate biocompatible and biodegradable scaffolds for skeletal muscle tissue engineering have increased significantly [211]. Three-dimensional bioprinting enables the layer-by-layer creation of scaffolds with an anatomically realistic reconstruction of the desired tissue structure, which can also control cellular alignment. Recently, decellularized ECM methacrylate (dECM-MA) from porcine skeletal muscles was used as a bioink and combined with fibrillated PVA to produce a uniaxially oriented patterned structure using 3D bioprinting [212]. Thereby, tissue-specific biochemical cues of the dECM-MA were combined with topographical cues. The incorporated myoblasts (C2C12 cell line) aligned and resulted in a high degree of myotube formation. Moreover, compared to GelMA scaffolds with the same topographical cues, dECM-based scaffolds resulted in the increased expression of myogenic genes (MyoD1, MYH2, and MyoG). Choi et al. produced dECM sponges, dECM hydrogels, and 3D cell printed muscle constructs using skeletal muscle dECM bioink and a granule-based printing reservoir [213]. The 3D bioprinted muscle constructs maintained high cell viability without creating a hypoxic environment (Figure 6A), exhibited densely packed and aligned myotubes throughout the construct (Figure 6B), and promoted de novo muscle formation in a VML rat model. Furthermore, by using coaxial nozzle printing with muscle and vascular dECM bioinks, prevascularized muscle constructs were generated that resulted in enhanced de novo muscle fiber formation, vascularization, innervation, and 85% functional recovery in VML injury. In 3D printing technology, the proper selection of nozzle size and extrusion pressure has also been shown to be effective for cell orientation [214]. Using an extrusion bioprinting approach, Distler and colleagues showed the rise of shear force by increasing the extrusion pressure and decreasing the diameter of the extrusion nozzle, and the frequency of F-actin orientation in the direction of 3D printed oxidized alginate–gelatin hydrogel was increased (Figure 6C,D). In addition, the orientation of the C2C12 cells decreased when the diameter of the extrusion nozzle remained the same and the pressure was decreased.

Kim et al. produced bundled PCL collagen-coated microfiber scaffolds using a microfibrillation/leaching process for the regeneration of muscle tissue [215]. The fibrillated PVA was leached from a PVA/PCL mixture to obtain the uniaxially aligned PCL bundles. The results demonstrated that C2C12 cells were well aligned along the direction of microfibrils and the myogenic differentiation was detected by MHC expression.

## 7. Conclusions

In summary, skeletal muscle tissue engineering offers promising perspectives in regenerative medicine. The ability to generate patient-derived iPSCs and the subsequent differentiation of these cells into myoblasts has great potential to overcome the limitations of producing autologous constructs. These myoblasts could enable the efficient regeneration of skeletal muscle tissue and prevent rejection reactions since the cells are autologous. In addition, the creation of innovative biomaterial scaffolds with distinct topography for cell guidance and 3D bioprinting technologies will enable the proper alignment and maturation of cells in an ECM-replicating environment. Further developments in skeletal muscle tissue engineering will provide innovative treatment options for patients with skeletal muscle loss due to injury, aging, and degeneration.

## Data Availability

Not applicable.

## References

[1] Qazi T.H., Mooney D.J., Pumberger M., Geissler S., Duda G.N. (2015). Biomaterials based strategies for skeletal muscle tissue engineering: Existing technologies and future trends. Biomaterials.

[2] Liu J., Saul D., Boker K.O., Ernst J., Lehman W., Schilling A.F. (2018). Current Methods for Skeletal Muscle Tissue Repair and Regeneration. Biomed. Res. Int..

[3] Carnes M.E., Pins G.D. (2020). Skeletal Muscle Tissue Engineering: Biomaterials-Based Strategies for the Treatment of Volumetric Muscle Loss. Bioengineering.

[4] Forcina L., Cosentino M., Musaro A. (2020). Mechanisms Regulating Muscle Regeneration: Insights into the Interrelated and Time-Dependent Phases of Tissue Healing. Cells.

[5] Basurto I.M., Mora M.T., Gardner G.M., Christ G.J., Caliari S.R. (2021). Aligned and Conductive 3D Collagen Scaffolds for Skeletal Muscle Tissue Engineering. Biomater. Sci..

[6] Cezar C.A., Mooney D.J. (2015). Biomaterial-based delivery for skeletal muscle repair. Adv. Drug Deliv. Rev..

[7] Xing F., Li L., Zhou C., Long C., Wu L., Lei H., Kong Q., Fan Y., Xiang Z., Zhang X. (2019). Regulation and Directing Stem Cell Fate by Tissue Engineering Functional Microenvironments: Scaffold Physical and Chemical Cues. Stem Cells Int..

[8] Smoak M.M., Mikos A.G. (2020). Advances in biomaterials for skeletal muscle engineering and obstacles still to overcome. Mater. Today Bio..

[9] Jarvinen T.A., Jarvinen M., Kalimo H. (2013). Regeneration of injured skeletal muscle after the injury. Muscles Ligaments Tendons J..

[10] Gilbert-Honick J., Iyer S.R., Somers S.M., Lovering R.M., Wagner K., Mao H.-Q., Grayson W.L. (2018). Engineering functional and histological regeneration of vascularized skeletal muscle. Biomaterials.

[11] Kim J.H., Kim I., Seol Y.-J., Ko I.K., Yoo J.J., Atala A., Lee S.J. (2020). Neural cell integration into 3D bioprinted skeletal muscle constructs accelerates restoration of muscle function. Nat. Commun..

[12] Yang W., Hu P. (2018). Skeletal muscle regeneration is modulated by inflammation. J. Orthop. Transl..

[13] McCullen S.D., Chow A.G.Y., Stevens M.M. (2011). In vivo tissue engineering of musculoskeletal tissues. Curr. Opin. Biotechnol..

[14] Russell C.S., Mostafavi A., Quint J.P., Panayi A.C., Baldino K., Williams T.J., Daubendiek J.G., Hugo Sánchez V., Bonick Z., Trujillo-Miranda M. (2020). In situ printing of adhesive hydrogel scaffolds for the treatment of skeletal muscle injuries. ACS Appl. Bio. Mater..

[15] Qazi T.H., Duda G.N., Ort M.J., Perka C., Geissler S., Winkler T. (2019). Cell therapy to improve regeneration of skeletal muscle injuries. J. Cachexia Sarcopenia Muscle.

[16] Pantelic M.N., Larkin L.M. (2018). Stem cells for skeletal muscle tissue engineering. Tissue Eng. Part. B Rev..

[17] McCullagh K.J.A., Perlingeiro R.C.R. (2015). Coaxing stem cells for skeletal muscle repair. Adv. Drug Deliv. Rev..

[18] Collins C.A., Olsen I., Zammit P.S., Heslop L., Petrie A., Partridge T.A., Morgan J.E. (2005). Stem cell function, self-renewal, and behavioral heterogeneity of cells from the adult muscle satellite cell niche. Cell.

[19] Dunn A., Talovic M., Patel K., Patel A., Marcinczyk M., Garg K. (2019). Biomaterial and stem cell-based strategies for skeletal muscle regeneration. J. Orthop. Res..

[20] Relaix F., Zammit P.S. (2012). Satellite cells are essential for skeletal muscle regeneration: The cell on the edge returns centre stage. Development.

[21] Yin H., Price F., Rudnicki M.A. (2013). Satellite cells and the muscle stem cell niche. Physiol. Rev..

[22] Tedesco F.S., Dellavalle A., Diaz-Manera J., Messina G., Cossu G. (2010). Repairing skeletal muscle: Regenerative potential of skeletal muscle stem cells. J. Clin. Investig..

[23] Fishman J.M., Tyraskis A., Maghsoudlou P., Urbani L., Totonelli G., Birchall M.A., De Coppi P. (2013). Skeletal muscle tissue engineering: Which cell to use?. Tissue Eng. Part B Rev..

[24] Yablonka-Reuveni Z. (2011). The skeletal muscle satellite cell: Still young and fascinating at 50. J. Histochem. Cytochem..

[25] Le Grand F., Rudnicki M.A. (2007). Skeletal muscle satellite cells and adult myogenesis. Curr. Opin. Cell Biol..

[26] Motohashi N., Asakura Y., Asakura A. (2014). Isolation, culture, and transplantation of muscle satellite cells. J. Vis. Exp. JOVE.

[27] Grefte S., Kuijpers-Jagtman A.M., Torensma R., Von den Hoff J.W. (2007). Skeletal muscle development and regeneration. Stem Cells Dev..

[28] Rinaldi F., Perlingeiro R.C.R. (2014). Stem cells for skeletal muscle regeneration: Therapeutic potential and roadblocks. Transl. Res..

[29] Sass F.A., Fuchs M., Pumberger M., Geissler S., Duda G.N., Perka C., Schmidt-Bleek K. (2018). Immunology guides skeletal muscle regeneration. Int. J. Mol. Sci..

[30] Judson R.N., Rossi F.M.V. (2020). Towards stem cell therapies for skeletal muscle repair. NPJ Regen. Med..

[31] Murphy M.M., Lawson J.A., Mathew S.J., Hutcheson D.A., Kardon G. (2011). Satellite cells, connective tissue fibroblasts and their interactions are crucial for muscle regeneration. Development.

[32] Czajka C.A., Calder B.W., Yost M.J., Drake C.J. (2015). Implanted scaffold-free prevascularized constructs promote tissue repair. Ann. Plast. Surg..

[33] Montarras D., Morgan J., Collins C., Relaix F., Zaffran S., Cumano A., Partridge T., Buckingham M. (2005). Direct isolation of satellite cells for skeletal muscle regeneration. Science.

[34] Sacco A., Doyonnas R., Kraft P., Vitorovic S., Blau H.M. (2008). Self-renewal and expansion of single transplanted muscle stem cells. Nature.

[35] Arpke R.W., Darabi R., Mader T.L., Zhang Y., Toyama A., Lonetree C.l., Nash N., Lowe D.A., Perlingeiro R.C.R., Kyba M. (2013). A New Immuno-, Dystrophin-Deficient Model, the NSG-mdx4Cv Mouse, Provides Evidence for Functional Improvement Following Allogeneic Satellite Cell Transplantation. Stem Cells.

[36] Morgan J.E., Watt D.J., Sloper J.C., Partridge T.A. (1988). Partial correction of an inherited biochemical defect of skeletal muscle by grafts of normal muscle precursor cells. J. Neurol. Sci..

[37] Watt D.J., Lambert K., Morgan J.E., Partridge T.A., Sloper J.C. (1982). Incorporation of donor muscle precursor cells into an area of muscle regeneration in the host mouse. J. Neurol. Sci..

[38] Partridge T.A., Morgan J.E., Coulton G.R., Hoffman E.P., Kunkel L.M. (1989). Conversion of mdx myofibres from dystrophin-negative to-positive by injection of normal myoblasts. Nature.

[39] Kinoshita I., Vilquin J.T., GuéRette B., Asselin I., Roy R., Tremblay J.P. (1994). Very efficient myoblast allotransplantation in mice under FK506 immunosuppression. Muscle Nerve Off. J. Am. Assoc. Electrodiagn. Med..

[40] Kinoshita I., Huard J., Tremblay J.P. (1994). Utilization of myoblasts from transgenic mice to evaluate the efficacy of myoblast transplantation. Muscle Nerve Off. J. Am. Assoc. Electrodiagn. Med..

[41] Huard J., Verreault S., Roy R., Tremblay M., Tremblay J.P. (1994). High efficiency of muscle regeneration after human myoblast clone transplantation in SCID mice. J. Clin. Investig..

[42] Rossi C.A., Flaibani M., Blaauw B., Pozzobon M., Figallo E., Reggiani C., Vitiello L., Elvassore N., De Coppi P. (2011). In vivo tissue engineering of functional skeletal muscle by freshly isolated satellite cells embedded in a photopolymerizable hydrogel. FASEB J..

[43] Prüller J., Mannhardt I., Eschenhagen T., Zammit P.S., Figeac N. (2018). Satellite cells delivered in their niche efficiently generate functional myotubes in three-dimensional cell culture. PLoS ONE.

[44] Gilbert P.M., Havenstrite K.L., Magnusson K.E.G., Sacco A., Leonardi N.A., Kraft P., Nguyen N.K., Thrun S., Lutolf M.P., Blau H.M. (2010). Substrate elasticity regulates skeletal muscle stem cell self-renewal in culture. Science.

[45] Pavlath G.K., Thaloor D., Rando T.A., Cheong M., English A.W., Zheng B. (1998). Heterogeneity among muscle precursor cells in adult skeletal muscles with differing regenerative capacities. Dev. Dyn. Off. Publ. Am. Assoc. Anat..

[46] Price F.D., Kuroda K., Rudnicki M.A. (2007). Stem cell based therapies to treat muscular dystrophy. Biochim. Biophys. Acta BBA Mol. Basis Dis..

[47] Shadrach J.L., Wagers A.J. (2011). Stem cells for skeletal muscle repair. Philos. Trans. R. Soc. B Biol. Sci..

[48] Péault B., Rudnicki M., Torrente Y., Cossu G., Tremblay J.P., Partridge T., Gussoni E., Kunkel L.M., Huard J. (2007). Stem and progenitor cells in skeletal muscle development, maintenance, and therapy. Mol. Ther..

[49] Karpati G., Ajdukovic D., Arnold D., Gledhill R.B., Guttmann R., Holland P., Koch P.A., Shoubridge E., Spence D., Vanasse M. (1993). Myoblast transfer in Duchenne muscular dystrophy. Ann. Neurol. Off. J. Am. Neurol. Assoc. Child. Neurol. Soc..

[50] Mendell J.R., Kissel J.T., Amato A.A., King W., Signore L., Prior T.W., Sahenk Z., Benson S., McAndrew P.E., Rice R. (1995). Myoblast transfer in the treatment of Duchenne’s muscular dystrophy. N. Engl. J. Med..

[51] Skuk D., Roy B., Goulet M., Tremblay J.P. (1999). Successful myoblast transplantation in primates depends on appropriate cell delivery and induction of regeneration in the host muscle. Exp. Neurol..

[52] Hill E., Boontheekul T., Mooney D.J. (2006). Designing scaffolds to enhance transplanted myoblast survival and migration. Tissue Eng..

[53] Machingal M.A., Corona B.T., Walters T.J., Kesireddy V., Koval C.N., Dannahower A., Zhao W., Yoo J.J., Christ G.J. (2011). A tissue-engineered muscle repair construct for functional restoration of an irrecoverable muscle injury in a murine model. Tissue Eng. Part A.

[54] Corona B.T., Machingal M.A., Criswell T., Vadhavkar M., Dannahower A.C., Bergman C., Zhao W., Christ G.J. (2012). Further development of a tissue engineered muscle repair construct in vitro for enhanced functional recovery following implantation in vivo in a murine model of volumetric muscle loss injury. Tissue Eng. Part A.

[55] Corona B.T., Ward C.L., Baker H.B., Walters T.J., Christ G.J. (2014). Implantation of in vitro tissue engineered muscle repair constructs and bladder acellular matrices partially restore in vivo skeletal muscle function in a rat model of volumetric muscle loss injury. Tissue Eng. Part A.

[56] Nakamura Y., Miyaki S., Ishitobi H., Matsuyama S., Nakasa T., Kamei N., Akimoto T., Higashi Y., Ochi M. (2015). Mesenchymal-stem-cell-derived exosomes accelerate skeletal muscle regeneration. FEBS Lett..

[57] Caplan A.I. (2005). Mesenchymal stem cells: Cell–based reconstructive therapy in orthopedics. Tissue Eng..

[58] Dezawa M., Ishikawa H., Itokazu Y., Yoshihara T., Hoshino M., Takeda S., Ide C., Nabeshima Y. (2005). Bone marrow stromal cells generate muscle cells and repair muscle degeneration. Science.

[59] Krampera M., Pizzolo G., Aprili G., Franchini M. (2006). Mesenchymal stem cells for bone, cartilage, tendon and skeletal muscle repair. Bone.

[60] Kern S., Eichler H., Stoeve J., Klüter H., Bieback K. (2006). Comparative analysis of mesenchymal stem cells from bone marrow, umbilical cord blood, or adipose tissue. Stem Cells.

[61] Mara C.S.d., Sartori A.R., Duarte A.S., Andrade A.L.L., Pedro M.A.C., Coimbra I.B. (2011). Periosteum as a source of mesenchymal stem cells: The effects of TGF-β3 on chondrogenesis. Clinics.

[62] Wang H.S., Hung S.C., Peng S.T., Huang C.C., Wei H.M., Guo Y.J., Fu Y.S., Lai M.C., Chen C.C. (2004). Mesenchymal stem cells in the Wharton’s jelly of the human umbilical cord. Stem Cells.

[63] Jacobs S.A., Roobrouck V.D., Verfaillie C.M., Van Gool S.W. (2013). Immunological characteristics of human mesenchymal stem cells and multipotent adult progenitor cells. Immunol. Cell Biol..

[64] Ankrum J.A., Ong J.F., Karp J.M. (2014). Mesenchymal stem cells: Immune evasive, not immune privileged. Nat. Biotechnol..

[65] Galli D., Vitale M., Vaccarezza M. (2014). Bone marrow-derived mesenchymal cell differentiation toward myogenic lineages: Facts and perspectives. Biomed. Res. Int..

[66] Sicari B.M., Dearth C.L., Badylak S.F. (2014). Tissue engineering and regenerative medicine approaches to enhance the functional response to skeletal muscle injury. Anat. Rec..

[67] Ferrari G., Angelis D., Coletta M., Paolucci E., Stornaiuolo A., Cossu G., Mavilio F. (1998). Muscle regeneration by bone marrow-derived myogenic progenitors. Science.

[68] Feng S.W., Lu X.L., Liu Z.S., Zhang Y.N., Liu T.Y., Li J.L., Yu M.J., Zeng Y., Zhang C. (2008). Dynamic distribution of bone marrow-derived mesenchymal stromal cells and change of pathology after infusing into mdx mice. Cytotherapy.

[69] Liu Q., Chen Z., Terry T., McNatt J.M., Willerson J.T., Zoldhelyi P. (2009). Intra-arterial transplantation of adult bone marrow cells restores blood flow and regenerates skeletal muscle in ischemic limbs. Vasc. Endovasc. Surg..

[70] Andrade B.M., Baldanza M.R., Ribeiro K.C., Porto A., Pecanha R., Fortes F.S.A., Zapata-Sudo G., Campos-de-Carvalho A.C., Goldenberg R.C.S., Werneck-de-Castro J.P. (2015). Bone marrow mesenchymal cells improve muscle function in a skeletal muscle re-injury model. PLoS ONE.

[71] Helal M.A.M., Shaheen N.E.M., Abu Zahra F.A. (2016). Immunomodulatory capacity of the local mesenchymal stem cells transplantation after severe skeletal muscle injury in female rats. Immunopharmacol. Immunotoxicol..

[72] Winkler T., von Roth P., Matziolis G., Mehta M., Perka C., Duda G.N. (2009). Dose–response relationship of mesenchymal stem cell transplantation and functional regeneration after severe skeletal muscle injury in rats. Tissue Eng. Part A.

[73] Maeda Y., Yonemochi Y., Nakajyo Y., Hidaka H., Ikeda T., Ando Y. (2017). CXCL12 and osteopontin from bone marrow-derived mesenchymal stromal cells improve muscle regeneration. Sci. Rep..

[74] Sassoli C., Vallone L., Tani A., Chellini F., Nosi D., Zecchi-Orlandini S. (2018). Combined use of bone marrow-derived mesenchymal stromal cells (BM-MSCs) and platelet rich plasma (PRP) stimulates proliferation and differentiation of myoblasts in vitro: New therapeutic perspectives for skeletal muscle repair/regeneration. Cell Tissue Res..

[75] Natsu K., Ochi M., Mochizuki Y., Hachisuka H., Yanada S., Yasunaga Y. (2004). Allogeneic bone marrow-derived mesenchymal stromal cells promote the regeneration of injured skeletal muscle without differentiation into myofibers. Tissue Eng..

[76] Merritt E.K., Cannon M.V., Hammers D.W., Le L.N., Gokhale R., Sarathy A., Song T.J., Tierney M.T., Suggs L.J., Walters T.J. (2010). Repair of traumatic skeletal muscle injury with bone-marrow-derived mesenchymal stem cells seeded on extracellular matrix. Tissue Eng. Part A.

[77] Kozlowska U., Krawczenko A., Futoma K., Jurek T., Rorat M., Patrzalek D., Klimczak A. (2019). Similarities and differences between mesenchymal stem/progenitor cells derived from various human tissues. World J. Stem Cells.

[78] Huri P.Y., Wang A., Spector A.A., Grayson W.L. (2014). Multistage adipose-derived stem cell myogenesis: An experimental and modeling study. Cell. Mol. Bioeng..

[79] Di Rocco G., Iachininoto M.G., Tritarelli A., Straino S., Zacheo A., Germani A., Crea F., Capogrossi M.C. (2006). Myogenic potential of adipose-tissue-derived cells. J. Cell Sci..

[80] da Justa Pinheiro C.H., de Queiroz J.C.F., Guimarães-Ferreira L., Vitzel K.F., Nachbar R.T., de Sousa L.G.O., de Souza-Jr A.L., Nunes M.T., Curi R. (2012). Local injections of adipose-derived mesenchymal stem cells modulate inflammation and increase angiogenesis ameliorating the dystrophic phenotype in dystrophin-deficient skeletal muscle. Stem Cell Rev. Rep..

[81] Kesireddy V. (2016). Evaluation of adipose-derived stem cells for tissue-engineered muscle repair construct-mediated repair of a murine model of volumetric muscle loss injury. Int. J. Nanomed..

[82] Meligy F.Y., Shigemura K., Behnsawy H.M., Fujisawa M., Kawabata M., Shirakawa T. (2012). The efficiency of in vitro isolation and myogenic differentiation of MSCs derived from adipose connective tissue, bone marrow, and skeletal muscle tissue. Vitr. Cell. Dev. Biol. Anim..

[83] Cao B., Zheng B., Jankowski R.J., Kimura S., Ikezawa M., Deasy B., Cummins J., Epperly M., Qu-Petersen Z., Huard J. (2003). Muscle stem cells differentiate into haematopoietic lineages but retain myogenic potential. Nat. Cell Biol..

[84] Lee J.Y., Qu-Petersen Z., Cao B., Kimura S., Jankowski R., Cummins J., Usas A., Gates C., Robbins P., Wernig A. (2000). Clonal isolation of muscle-derived cells capable of enhancing muscle regeneration and bone healing. J. Cell Biol..

[85] Bhagavati S. (2008). Stem cell based therapy for skeletal muscle diseases. Curr. Stem Cell Res. Ther..

[86] Matthias N., Hunt S.D., Wu J., Lo J., Callahan L.A.S., Li Y., Huard J., Darabi R. (2018). Volumetric muscle loss injury repair using in situ fibrin gel cast seeded with muscle-derived stem cells (MDSCs). Stem Cell Res..

[87] Usas A., Huard J. (2007). Muscle-derived stem cells for tissue engineering and regenerative therapy. Biomaterials.

[88] Deasy B.M., Jankowski R.J., Huard J. (2001). Muscle-derived stem cells: Characterization and potential for cell-mediated therapy. Blood Cells Mol. Dis..

[89] Torrente Y., Tremblay J.P., Pisati F., Belicchi M., Rossi B., Sironi M., Fortunato F., El Fahime M., D’Angelo M.G., Caron N.J. (2001). Intraarterial injection of muscle-derived CD34+Sca-1+ stem cells restores dystrophin in mdx mice. J. Cell Biol..

[90] Qu-Petersen Z., Deasy B., Jankowski R., Ikezawa M., Cummins J., Pruchnic R., Mytinger J., Cao B., Gates C., Wernig A. (2002). Identification of a novel population of muscle stem cells in mice potential for muscle regeneration. J. Cell Biol..

[91] Rouger K., Larcher T., Dubreil L., Deschamps J.-Y., Le Guiner C., Jouvion G., Delorme B., Lieubeau B., Carlus M., Fornasari B. (2011). Systemic delivery of allogenic muscle stem cells induces long-term muscle repair and clinical efficacy in duchenne muscular dystrophy dogs. Am. J. Pathol..

[92] Lorant J., Saury C., Schleder C., Robriquet F., Lieubeau B., Négroni E., Leroux I., Chabrand L., Viau S., Babarit C. (2018). Skeletal muscle regenerative potential of human MuStem cells following transplantation into injured mice muscle. Mol. Ther..

[93] Skuk D., Goulet M., Roy B., Piette V., Côté C.H., Chapdelaine P., Hogrel J.-Y., Paradis M., Bouchard J.-P., Sylvain M. (2007). First test of a “high-density injection” protocol for myogenic cell transplantation throughout large volumes of muscles in a Duchenne muscular dystrophy patient: Eighteen months follow-up. Neuromuscul. Disord..

[94] Klimczak A., Zimna A., Malcher A., Kozlowska U., Futoma K., Czarnota J., Kemnitz P., Bryl A., Kurpisz M. (2020). Co-Transplantation of Bone Marrow-MSCs and Myogenic Stem/Progenitor Cells from Adult Donors Improves Muscle Function of Patients with Duchenne Muscular Dystrophy. Cells.

[95] Farini A., Razini P., Erratico S., Torrente Y., Meregalli M. (2009). Cell based therapy for Duchenne muscular dystrophy. J. Cell. Physiol..

[96] Dellavalle A., Sampaolesi M., Tonlorenzi R., Tagliafico E., Sacchetti B., Perani L., Innocenzi A., Galvez B.G., Messina G., Morosetti R. (2007). Pericytes of human skeletal muscle are myogenic precursors distinct from satellite cells. Nat. Cell Biol..

[97] Birbrair A., Delbono O. (2015). Pericytes are essential for skeletal muscle formation. Stem Cell Rev. Rep..

[98] Dellavalle A., Maroli G., Covarello D., Azzoni E., Innocenzi A., Perani L., Antonini S., Sambasivan R., Brunelli S., Tajbakhsh S. (2011). Pericytes resident in postnatal skeletal muscle differentiate into muscle fibres and generate satellite cells. Nat. Commun..

[99] Birbrair A., Zhang T., Wang Z.-M., Messi M.L., Enikolopov G.N., Mintz A., Delbono O. (2013). Role of pericytes in skeletal muscle regeneration and fat accumulation. Stem Cells Dev..

[100] Fuoco C., Sangalli E., Vono R., Testa S., Sacchetti B., Latronico M.V., Bernardini S., Madeddu P., Cesareni G., Seliktar D. (2014). 3D hydrogel environment rejuvenates aged pericytes for skeletal muscle tissue engineering. Front. Physiol..

[101] Takahashi K., Tanabe K., Ohnuki M., Narita M., Ichisaka T., Tomoda K., Yamanaka S. (2007). Induction of pluripotent stem cells from adult human fibroblasts by defined factors. Cell.

[102] van der Wal E., Herrero-Hernandez P., Wan R., Broeders M., In’t Groen S.L.M., van Gestel T.J.M., van Ijcken W.F.J., Cheung T.H., van der Ploeg A.T., Schaaf G.J. (2018). Large-scale expansion of human iPSC-derived skeletal muscle cells for disease modeling and cell-based therapeutic strategies. Stem Cell Rep..

[103] Steinle H., Weber M., Behring A., Mau-Holzmann U., von Ohle C., Popov A.F., Schlensak C., Wendel H.P., Avci-Adali M. (2019). Reprogramming of Urine-Derived Renal Epithelial Cells into iPSCs Using srRNA and Consecutive Differentiation into Beating Cardiomyocytes. Mol. Nucleic Acids.

[104] Del Carmen Ortuno-Costela M., Garcia-Lopez M., Cerrada V., Gallardo M.E. (2019). iPSCs: A powerful tool for skeletal muscle tissue engineering. J. Cell Mol. Med..

[105] Ben-David U., Benvenisty N. (2011). The tumorigenicity of human embryonic and induced pluripotent stem cells. Nat. Rev. Cancer.

[106] Roca I., Requena J., Edel M.J., Alvarez-Palomo A.B. (2015). Myogenic precursors from iPS cells for skeletal muscle cell replacement therapy. J. Clin. Med..

[107] Choi I.Y., Lim H., Estrellas K., Mula J., Cohen T.V., Zhang Y., Donnelly C.J., Richard J.-P., Kim Y.J., Kim H. (2016). Concordant but varied phenotypes among Duchenne muscular dystrophy patient-specific myoblasts derived using a human iPSC-based model. Cell Rep..

[108] Chal J., Al Tanoury Z., Hestin M., Gobert B., Aivio S., Hick A., Cherrier T., Nesmith A.P., Parker K.K., Pourquié O. (2016). Generation of human muscle fibers and satellite-like cells from human pluripotent stem cells in vitro. Nat. Protoc..

[109] Goudenege S., Lebel C., Huot N.B., Dufour C., Fujii I., Gekas J., Rousseau J., Tremblay J.P. (2012). Myoblasts derived from normal hESCs and dystrophic hiPSCs efficiently fuse with existing muscle fibers following transplantation. Mol. Ther..

[110] Mizuno Y., Chang H., Umeda K., Niwa A., Iwasa T., Awaya T., Fukada S.i., Yamamoto H., Yamanaka S., Nakahata T. (2010). Generation of skeletal muscle stem/progenitor cells from murine induced pluripotent stem cells. FASEB J..

[111] Rao L., Qian Y., Khodabukus A., Ribar T., Bursac N. (2018). Engineering human pluripotent stem cells into a functional skeletal muscle tissue. Nat. Commun..

[112] Maffioletti S.M., Sarcar S., Henderson A.B.H., Mannhardt I., Pinton L., Moyle L.A., Steele-Stallard H., Cappellari O., Wells K.E., Ferrari G. (2018). Three-dimensional human iPSC-derived artificial skeletal muscles model muscular dystrophies and enable multilineage tissue engineering. Cell Rep..

[113] Darabi R., Pan W., Bosnakovski D., Baik J., Kyba M., Perlingeiro R.C.R. (2011). Functional myogenic engraftment from mouse iPS cells. Stem Cell Rev. Rep..

[114] Darabi R., Arpke R.W., Irion S., Dimos J.T., Grskovic M., Kyba M., Perlingeiro R.C.R. (2012). Human ES-and iPS-derived myogenic progenitors restore DYSTROPHIN and improve contractility upon transplantation in dystrophic mice. Cell Stem Cell.

[115] Shoji E., Woltjen K., Sakurai H. (2016). Directed myogenic differentiation of human induced pluripotent stem cells. Methods Mol. Biol..

[116] Hosoyama T., McGivern J.V., Van Dyke J.M., Ebert A.D., Suzuki M. (2014). Derivation of myogenic progenitors directly from human pluripotent stem cells using a sphere-based culture. Stem Cells Transl. Med..

[117] Jiwlawat S., Lynch E., Glaser J., Smit-Oistad I., Jeffrey J., Van Dyke J.M., Suzuki M. (2017). Differentiation and sarcomere formation in skeletal myocytes directly prepared from human induced pluripotent stem cells using a sphere-based culture. Differentiation.

[118] Borchin B., Chen J., Barberi T. (2013). Derivation and FACS-mediated purification of PAX3+/PAX7+ skeletal muscle precursors from human pluripotent stem cells. Stem Cell Rep..

[119] van der Wal E., Bergsma A.J., Van Gestel T.J.M., Lm S., Zaehres H., Araúzo-Bravo M.J., Schöler H.R., van der Ploeg A.T., Pijnappel W.W.M.P. (2017). GAA deficiency in Pompe disease is alleviated by exon inclusion in iPSC-derived skeletal muscle cells. Mol. Ther. Nucleic Acids.

[120] Baci D., Chirivì M., Pace V., Maiullari F., Milan M., Rampin A., Somma P., Presutti D., Garavelli S., Bruno A. (2020). Extracellular Vesicles from Skeletal Muscle Cells Efficiently Promote Myogenesis in Induced Pluripotent Stem Cells. Cells.

[121] Zhou L., Ge J., Wang M., Chen M., Cheng W., Ji W., Lei B. (2020). Injectable muscle-adhesive antioxidant conductive photothermal bioactive nanomatrix for efficiently promoting full-thickness skeletal muscle regeneration. Bioact. Mater..

[122] Lev R., Seliktar D. (2018). Hydrogel biomaterials and their therapeutic potential for muscle injuries and muscular dystrophies. J. R. Soc. Interface.

[123] Mei X., Cheng K. (2020). Recent Development in Therapeutic Cardiac Patches. Front. Cardiovasc. Med..

[124] Nakayama K.H., Shayan M., Huang N.F. (2019). Engineering biomimetic materials for skeletal muscle repair and regeneration. Adv. Healthc. Mater..

[125] Fischer K.M., Scott T.E., Browe D.P., McGaughey T.A., Wood C., Wolyniak M.J., Freeman J.W. (2020). Hydrogels for Skeletal Muscle Regeneration. Regen. Eng. Transl. Med..

[126] Camci-Unal G., Annabi N., Dokmeci M.R., Liao R., Khademhosseini A. (2014). Hydrogels for cardiac tissue engineering. NPG Asia Mater..

[127] McLaughlin S., McNeill B., Podrebarac J., Hosoyama K., Sedlakova V., Cron G., Smyth D., Seymour R., Goel K., Liang W. (2019). Injectable human recombinant collagen matrices limit adverse remodeling and improve cardiac function after myocardial infarction. Nat. Commun..

[128] Feng M., Liu X., Hou X., Chen J., Zhang H., Song S., Han X., Shi C. (2020). Specific angiogenic peptide binding with injectable cardiac ECM collagen gel promotes the recovery of myocardial infarction in rat. J. Biomed. Mater. Res. Part. A.

[129] Le L.V., Mohindra P., Fang Q., Sievers R.E., Mkrtschjan M.A., Solis C., Safranek C.W., Russell B., Lee R.J., Desai T.A. (2018). Injectable hyaluronic acid based microrods provide local micromechanical and biochemical cues to attenuate cardiac fibrosis after myocardial infarction. Biomaterials.

[130] Stilhano R.S., Madrigal J.L., Wong K., Williams P.A., Martin P.K., Yamaguchi F.S., Samoto V.Y., Han S.W., Silva E.A. (2016). Injectable alginate hydrogel for enhanced spatiotemporal control of lentivector delivery in murine skeletal muscle. J. Control. Release.

[131] Fang R., Tian W., Chen X. (2017). Synthesis of injectable alginate hydrogels with muscle-derived stem cells for potential myocardial infarction repair. Appl. Sci..

[132] Feng J., Wu Y., Chen W., Li J., Wang X., Chen Y., Yu Y., Shen Z., Zhang Y. (2020). Sustained release of bioactive IGF-1 from a silk fibroin microsphere-based injectable alginate hydrogel for the treatment of myocardial infarction. J. Mater. Chem. B.

[133] Liu J., Xu H.H., Zhou H., Weir M.D., Chen Q., Trotman C.A. (2013). Human umbilical cord stem cell encapsulation in novel macroporous and injectable fibrin for muscle tissue engineering. Acta Biomater..

[134] Melly L., Grosso A., Stanciu Pop C., Yu-Hsuan C., Nollevaux M.C., Schachtrup C., Marsano A., Di Maggio N., Rondelet B., Banfi A. (2020). Fibrin hydrogels promote scar formation and prevent therapeutic angiogenesis in the heart. J. Tissue Eng. Regen. Med..

[135] Guo B., Qu J., Zhao X., Zhang M. (2019). Degradable conductive self-healing hydrogels based on dextran-graft-tetraaniline and N-carboxyethyl chitosan as injectable carriers for myoblast cell therapy and muscle regeneration. Acta Biomater..

[136] Pollot B.E., Rathbone C.R., Wenke J.C., Guda T. (2018). Natural polymeric hydrogel evaluation for skeletal muscle tissue engineering. J. Biomed. Mater. Res. Part B Appl. Biomater..

[137] Boso D., Maghin E., Carraro E., Giagante M., Pavan P., Piccoli M. (2020). Extracellular matrix-derived hydrogels as biomaterial for different skeletal muscle tissue replacements. Materials.

[138] Theus A.S., Tomov M.L., Cetnar A., Lima B., Nish J., McCoy K., Mahmoudi M., Serpooshan V. (2019). Biomaterial approaches for cardiovascular tissue engineering. Emergent Mater..

[139] Tashakori-Miyanroudi M., Rakhshan K., Ramez M., Asgarian S., Janzadeh A., Azizi Y., Seifalian A., Ramezani F. (2020). Conductive carbon nanofibers incorporated into collagen bio-scaffold assists myocardial injury repair. Int. J. Biol. Macromol..

[140] Pagliarosi O., Picchio V., Chimenti I., Messina E., Gaetani R. (2020). Building an artificial cardiac microenvironment: A focus on the extracellular matrix. Front. Cell Dev. Biol..

[141] Mao M., He J., Li Z., Han K., Li D. (2020). Multi-directional cellular alignment in 3D guided by electrohydrodynamically-printed microlattices. Acta Biomater..

[142] Li M.-T., Ruehle M.A., Stevens H.Y., Servies N., Willett N.J., Karthikeyakannan S., Warren G.L., Guldberg R.E., Krishnan L. (2017). Skeletal myoblast-seeded vascularized tissue scaffolds in the treatment of a large volumetric muscle defect in the rat biceps femoris muscle. Tissue Eng. Part A.

[143] Gupta D., Santoso J.W., McCain M.L. (2021). Characterization of Gelatin Hydrogels Cross-Linked with Microbial Transglutaminase as Engineered Skeletal Muscle Substrates. Bioengineering.

[144] Bettadapur A., Suh G.C., Geisse N.A., Wang E.R., Hua C., Huber H.A., Viscio A.A., Kim J.Y., Strickland J.B., McCain M.L. (2016). Prolonged Culture of Aligned Skeletal Myotubes on Micromolded Gelatin Hydrogels. Sci. Rep..

[145] Wang L., Wu Y., Guo B., Ma P.X. (2015). Nanofiber yarn/hydrogel core–shell scaffolds mimicking native skeletal muscle tissue for guiding 3D myoblast alignment, elongation, and differentiation. ACS Nano.

[146] Broer T., Khodabukus A., Bursac N. (2020). Can we mimic skeletal muscles for novel drug discovery?. Expert Opin. Drug Discov..

[147] Marcinczyk M., Elmashhady H., Talovic M., Dunn A., Bugis F., Garg K. (2017). Laminin-111 enriched fibrin hydrogels for skeletal muscle regeneration. Biomaterials.

[148] Gholobova D., Terrie L., Gerard M., Declercq H., Thorrez L. (2020). Vascularization of tissue-engineered skeletal muscle constructs. Biomaterials.

[149] Yi H., Forsythe S., He Y., Liu Q., Xiong G., Wei S., Li G., Atala A., Skardal A., Zhang Y. (2017). Tissue-specific extracellular matrix promotes myogenic differentiation of human muscle progenitor cells on gelatin and heparin conjugated alginate hydrogels. Acta Biomater..

[150] Majid Q.A., Fricker A.T., Gregory D.A., Davidenko N., Hernandez Cruz O., Jabbour R.J., Owen T.J., Basnett P., Lukasiewicz B., Stevens M. (2020). Natural Biomaterials for Cardiac Tissue Engineering: A Highly Biocompatible Solution. Front. Cardiovasc. Med..

[151] Ansari S., Chen C., Xu X., Annabi N., Zadeh H.H., Wu B.M., Khademhosseini A., Shi S., Moshaverinia A. (2016). Muscle tissue engineering using gingival mesenchymal stem cells encapsulated in alginate hydrogels containing multiple growth factors. Ann. Biomed. Eng..

[152] Kwee B.J., Mooney D.J. (2017). Biomaterials for skeletal muscle tissue engineering. Curr. Opin. Biotechnol..

[153] Boontheekul T., Hill E.E., Kong H.-J., Mooney D.J. (2007). Regulating myoblast phenotype through controlled gel stiffness and degradation. Tissue Eng..

[154] Yeo M., Lee H., Kim G.H. (2016). Combining a micro/nano-hierarchical scaffold with cell-printing of myoblasts induces cell alignment and differentiation favorable to skeletal muscle tissue regeneration. Biofabrication.

[155] Rico P., Rodrigo-Navarro A., Salmerón-Sánchez M. (2015). Borax-loaded PLLA for promotion of myogenic differentiation. Tissue Eng. Part A.

[156] Levenberg S., Rouwkema J., Macdonald M., Garfein E.S., Kohane D.S., Darland D.C., Marini R., Van Blitterswijk C.A., Mulligan R.C., D’Amore P.A. (2005). Engineering vascularized skeletal muscle tissue. Nat. Biotechnol..

[157] Wolf M.T., Dearth C.L., Sonnenberg S.B., Loboa E.G., Badylak S.F. (2015). Naturally derived and synthetic scaffolds for skeletal muscle reconstruction. Adv. Drug Deliv. Rev..

[158] Ostrovidov S., Salehi S., Costantini M., Suthiwanich K., Ebrahimi M., Sadeghian R.B., Fujie T., Shi X., Cannata S., Gargioli C. (2019). 3D bioprinting in skeletal muscle tissue engineering. Small.

[159] Apsite I., Uribe J.M., Posada A.F., Rosenfeldt S., Salehi S., Ionov L. (2019). 4D biofabrication of skeletal muscle microtissues. Biofabrication.

[160] Kook Y.-M., Hwang S., Kim H., Rhee K.-J., Lee K., Koh W.-G. (2020). Cardiovascular tissue regeneration system based on multiscale scaffolds comprising double-layered hydrogels and fibers. Sci. Rep..

[161] Salimath A.S., García A.J. (2016). Biofunctional hydrogels for skeletal muscle constructs. J. Tissue Eng. Regen. Med..

[162] Han W.M., Mohiuddin M., Anderson S.E., García A.J., Jang Y.C. (2019). Co-delivery of Wnt7a and muscle stem cells using synthetic bioadhesive hydrogel enhances murine muscle regeneration and cell migration during engraftment. Acta Biomater..

[163] Han W.M., Anderson S.E., Mohiuddin M., Barros D., Nakhai S.A., Shin E., Amaral I.F., Pêgo A.P., García A.J., Jang Y.C. (2018). Synthetic matrix enhances transplanted satellite cell engraftment in dystrophic and aged skeletal muscle with comorbid trauma. Sci. Adv..

[164] Xie M., Wang L., Guo B., Wang Z., Chen Y.E., Ma P.X. (2015). Ductile electroactive biodegradable hyperbranched polylactide copolymers enhancing myoblast differentiation. Biomaterials.

[165] Riboldi S.A., Sampaolesi M., Neuenschwander P., Cossu G., Mantero S. (2005). Electrospun degradable polyesterurethane membranes: Potential scaffolds for skeletal muscle tissue engineering. Biomaterials.

[166] Naureen B., Haseeb A., Basirun W., Muhamad F. (2021). Recent advances in tissue engineering scaffolds based on polyurethane and modified polyurethane. Mater. Sci. Eng. C.

[167] Ergene E., Yagci B.S., Gokyer S., Eyidogan A., Aksoy E.A., Huri P.Y. (2019). A novel polyurethane-based biodegradable elastomer as a promising material for skeletal muscle tissue engineering. Biomed. Mater..

[168] Jamadi E.S., Ghasemi-Mobarakeh L., Morshed M., Sadeghi M., Prabhakaran M.P., Ramakrishna S. (2016). Synthesis of polyester urethane urea and fabrication of elastomeric nanofibrous scaffolds for myocardial regeneration. Mater. Sci. Eng. C.

[169] Vannozzi L., Ricotti L., Santaniello T., Terencio T., Oropesa-Nunez R., Canale C., Borghi F., Menciassi A., Lenardi C., Gerges I. (2017). 3D porous polyurethanes featured by different mechanical properties: Characterization and interaction with skeletal muscle cells. J. Mech. Behav. Biomed. Mater..

[170] Sun Y., Duffy R., Lee A., Feinberg A.W. (2013). Optimizing the structure and contractility of engineered skeletal muscle thin films. Acta Biomater..

[171] Browe D.P., Wood C., Sze M.T., White K.A., Scott T., Olabisi R.M., Freeman J.W. (2017). Characterization and optimization of actuating poly (ethylene glycol) diacrylate/acrylic acid hydrogels as artificial muscles. Polymer.

[172] Jo H., Sim M., Kim S., Yang S., Yoo Y., Park J.-H., Yoon T.H., Kim M.-G., Lee J.Y. (2017). Electrically conductive graphene/polyacrylamide hydrogels produced by mild chemical reduction for enhanced myoblast growth and differentiation. Acta Biomater..

[173] Hosseinzadeh S., Rezayat S.M., Giaseddin A., Aliyan A., Soleimani M. (2018). Microfluidic system for synthesis of nanofibrous conductive hydrogel and muscle differentiation. J. Biomater. Appl..

[174] Jun I., Jeong S., Shin H. (2009). The stimulation of myoblast differentiation by electrically conductive sub-micron fibers. Biomaterials.

[175] Fallahi A., Yazdi I.K., Serex L., Lesha E., Faramarzi N., Tarlan F., Avci H., Costa-Almeida R., Sharifi F., Rinoldi C. (2019). Customizable composite fibers for engineering skeletal muscle models. Acs Biomater. Sci. Eng..

[176] García-Lizarribar A., Fernández-Garibay X., Velasco-Mallorquí F., Castaño A.G., Samitier J., Ramon-Azcon J. (2018). Composite Biomaterials as Long-Lasting Scaffolds for 3D Bioprinting of Highly Aligned Muscle Tissue. Macromol. Biosci..

[177] Noh S., Gong H.Y., Lee H.J., Koh W.-G. (2021). Electrically Conductive Micropatterned Polyaniline-Poly (ethylene glycol) Composite Hydrogel. Materials.

[178] Ku S.H., Park C.B. (2013). Myoblast differentiation on graphene oxide. Biomaterials.

[179] Chaudhuri B., Mondal B., Kumar S., Sarkar S. (2016). Myoblast differentiation and protein expression in electrospun graphene oxide (GO)-poly (ε-caprolactone, PCL) composite meshes. Mater. Lett..

[180] Uehara T.M., Paino I.M., Santos F.A., Scagion V.P., Correa D.S., Zucolotto V. (2020). Fabrication of random and aligned electrospun nanofibers containing graphene oxide for skeletal muscle cells scaffold. Polym. Adv. Technol..

[181] Velasco-Mallorquí F., Fernández-Costa J.M., Neves L., Ramón-Azcón J. (2020). New volumetric CNT-doped gelatin–cellulose scaffolds for skeletal muscle tissue engineering. Nanoscale Adv..

[182] Patel A., Mukundan S., Wang W., Karumuri A., Sant V., Mukhopadhyay S.M., Sant S. (2016). Carbon-based hierarchical scaffolds for myoblast differentiation: Synergy between nano-functionalization and alignment. Acta Biomater..

[183] McCorry M.C., Ohlson C., Gunnel S., Higginbottom S., Billiar K., Page R. Mechanical stimulation device for skeletal muscle tissue engineering. Proceedings of the 2012 38th Annual Northeast Bioengineering Conference (NEBEC).

[184] Ten Broek R.W., Grefte S., Von den Hoff J.W. (2010). Regulatory factors and cell populations involved in skeletal muscle regeneration. J. Cell Physiol..

[185] Powell C.A., Smiley B.L., Mills J., Vandenburgh H.H. (2002). Mechanical stimulation improves tissue-engineered human skeletal muscle. Am. J. Physiol. Cell Physiol..

[186] Zanchi N.E., Lancha A.H. (2008). Mechanical stimuli of skeletal muscle: Implications on mTOR/p70s6k and protein synthesis. Eur. J. Appl. Physiol..

[187] Okano T., Matsuda T. (1998). Tissue engineered skeletal muscle: Preparation of highly dense, highly oriented hybrid muscular tissues. Cell Transplant..

[188] Auluck A., Mudera V., Hunt N.P., Lewis M.P. (2005). A three-dimensional in vitro model system to study the adaptation of craniofacial skeletal muscle following mechanostimulation. Eur. J. Oral. Sci..

[189] Sakiyama K., Abe S., Tamatsu Y., Ide Y. (2005). Effects of stretching stress on the muscle contraction proteins of skeletal muscle myoblasts. Biomed. Res..

[190] Zhang S.J., Truskey G.A., Kraus W.E. (2007). Effect of cyclic stretch on β1D-integrin expression and activation of FAK and RhoA. Am. J. Physiol. Cell Physiol..

[191] Moon D.G., Christ G., Stitzel J.D., Atala A., Yoo J.J. (2008). Cyclic mechanical preconditioning improves engineered muscle contraction. Tissue Eng. Part A.

[192] Egusa H., Kobayashi M., Matsumoto T., Sasaki J.-I., Uraguchi S., Yatani H. (2013). Application of Cyclic Strain for Accelerated Skeletal Myogenic Differentiation of Mouse Bone Marrow-Derived Mesenchymal Stromal Cells with Cell Alignment. Tissue Eng. Part A.

[193] Harrison R.G. (1911). On the stereotropism of embryonic cells. Science.

[194] Weiss P. (1945). Experiments on cell and axon orientation in vitro: The role of colloidal exudates in tissue organization. J. Exp. Zool..

[195] Li Y., Huang G., Zhang X., Wang L., Du Y., Lu T.J., Xu F. (2014). Engineering cell alignment in vitro. Biotechnol. Adv..

[196] Metavarayuth K., Sitasuwan P., Zhao X., Lin Y., Wang Q. (2016). Engineering. Influence of surface topographical cues on the differentiation of mesenchymal stem cells in vitro. ACS Biomater. Sci. Eng..

[197] Avci H., Ghorbanpoor H., Nurbas M. (2018). Preparation of origanum minutiflorum oil-loaded core–shell structured chitosan nanofibers with tunable properties. Polym. Bull..

[198] Avci H., Akkulak E., Gergeroglu H., Ghorbanpoor H., Uysal O., Eker Sariboyaci A., Demir B., Soykan M.N., Pat S., Mohammadigharehbagh R. (2020). Flexible poly (styrene-ethylene-butadiene-styrene) hybrid nanofibers for bioengineering and water filtration applications. J. Appl. Polym. Sci..

[199] Nemati S., Kim S.-j., Shin Y.M., Shin H. (2019). Current progress in application of polymeric nanofibers to tissue engineering. Nano Converg..

[200] Hajiali H., Contestabile A., Mele E., Athanassiou A. (2018). Influence of topography of nanofibrous scaffolds on functionality of engineered neural tissue. J. Mater. Chem. B.

[201] Huang N.F., Patel S., Thakar R.G., Wu J., Hsiao B.S., Chu B., Lee R.J., Li S. (2006). Myotube assembly on nanofibrous and micropatterned polymers. Nano Lett..

[202] Woods I., Black A., Jockenhoevel S., Flanagan T.C. (2019). Harnessing topographical & biochemical cues to enhance elastogenesis by paediatric cells for cardiovascular tissue engineering applications. Biochem. Biophys. Res. Commun..

[203] Tsai S.-W., Yu Y.-L., Hsu F.-Y. (2019). Fabrication of polycaprolactone tubular scaffolds with an orthogonal-bilayer structure for smooth muscle cells. Mater. Sci. Eng. C.

[204] Wu Y., Wang L., Guo B., Ma P.X. (2017). Interwoven aligned conductive nanofiber yarn/hydrogel composite scaffolds for engineered 3D cardiac anisotropy. ACS Nano.

[205] Hu Q., Su C., Zeng Z., Zhang H., Feng R., Feng J., Li S. (2020). Fabrication of multilayer tubular scaffolds with aligned nanofibers to guide the growth of endothelial cells. J. Biomater. Appl..

[206] Eom S., Park S.M., Lim J., Kim D.S. Electrospun random/aligned hybrid nanofiber mat for development of multi-layered cardiac muscle patch. Proceedings of the 2018 IEEE International Conference on Cyborg and Bionic Systems (CBS).

[207] McKeon-Fischer K.D., Flagg D.H., Freeman J.W. (2011). Coaxial electrospun poly (ε-caprolactone), multiwalled carbon nanotubes, and polyacrylic acid/polyvinyl alcohol scaffold for skeletal muscle tissue engineering. J. Biomed. Mater. Res. Part A.

[208] Hosseini V., Ahadian S., Ostrovidov S., Camci-Unal G., Chen S., Kaji H., Ramalingam M., Khademhosseini A. (2012). Engineered contractile skeletal muscle tissue on a microgrooved methacrylated gelatin substrate. Tissue Eng. Part A.

[209] Jiwlawat N., Lynch E.M., Napiwocki B.N., Stempien A., Ashton R.S., Kamp T.J., Crone W.C., Suzuki M. (2019). Micropatterned substrates with physiological stiffness promote cell maturation and Pompe disease phenotype in human induced pluripotent stem cell-derived skeletal myocytes. Biotechnol. Bioeng..

[210] Chen S., Nakamoto T., Kawazoe N., Chen G. (2015). Engineering multi-layered skeletal muscle tissue by using 3D microgrooved collagen scaffolds. Biomaterials.

[211] Capel A.J., Rimington R.P., Fleming J.W., Player D.J., Baker L.A., Turner M.C., Jones J.M., Martin N.R., Ferguson R.A., Mudera V.C. (2019). Scalable 3D printed molds for human tissue engineered skeletal muscle. Front. Bioeng. Biotechnol..

[212] Kim W., Lee H., Lee J., Atala A., Yoo J.J., Lee S.J., Kim G.H. (2020). Efficient myotube formation in 3D bioprinted tissue construct by biochemical and topographical cues. Biomaterials.

[213] Choi Y.-J., Jun Y.-J., Kim D.Y., Yi H.-G., Chae S.-H., Kang J., Lee J., Gao G., Kong J.-S., Jang J. (2019). A 3D cell printed muscle construct with tissue-derived bioink for the treatment of volumetric muscle loss. Biomaterials.

[214] Distler T., Sulistio A., Schneidereit D., Friedrich O., Detsch R., Boccaccini A.R. (2020). 3D printed oxidized alginate-gelatin bioink provides guidance for C2C12 muscle precursor cell orientation and differentiation via shear stress during bioprinting. Biofabrication.

[215] Kim W., Kim M., Kim G.H. (2018). 3D-printed biomimetic scaffold simulating microfibril muscle structure. Adv. Funct. Mater..

